# Cross-frequency coupling in cortico-hippocampal networks supports the maintenance of sequential auditory information in short-term memory

**DOI:** 10.1371/journal.pbio.3002512

**Published:** 2024-03-05

**Authors:** Arthur Borderie, Anne Caclin, Jean-Philippe Lachaux, Marcela Perrone-Bertollotti, Roxane S. Hoyer, Philippe Kahane, Hélène Catenoix, Barbara Tillmann, Philippe Albouy

**Affiliations:** 1 CERVO Brain Research Center, School of Psychology, Laval University, Québec, Canada; 2 International Laboratory for Brain, Music and Sound Research (BRAMS), CRBLM, Montreal, Canada; 3 Université Claude Bernard Lyon 1, CNRS, INSERM, Centre de Recherche en Neurosciences de Lyon CRNL U1028 UMR5292, Bron, France; 4 Univ. Grenoble Alpes, Univ. Savoie Mont Blanc, CNRS, LPNC, Grenoble, France; 5 Univ. Grenoble Alpes, Inserm, U1216, CHU Grenoble Alpes, Grenoble Institut Neurosciences, Grenoble, France; 6 Department of Functional Neurology and Epileptology, Lyon Civil Hospices, member of the ERN EpiCARE, and Lyon 1 University, Lyon, France; 7 Laboratory for Research on Learning and Development, LEAD–CNRS UMR5022, Université de Bourgogne, Dijon, France; Newcastle University Medical School, UNITED KINGDOM

## Abstract

It has been suggested that cross-frequency coupling in cortico-hippocampal networks enables the maintenance of multiple visuo-spatial items in working memory. However, whether this mechanism acts as a global neural code for memory retention across sensory modalities remains to be demonstrated. Intracranial EEG data were recorded while drug-resistant patients with epilepsy performed a delayed matched-to-sample task with tone sequences. We manipulated task difficulty by varying the memory load and the duration of the silent retention period between the to-be-compared sequences. We show that the strength of theta-gamma phase amplitude coupling in the superior temporal sulcus, the inferior frontal gyrus, the inferior temporal gyrus, and the hippocampus (i) supports the short-term retention of auditory sequences; (ii) decodes correct and incorrect memory trials as revealed by machine learning analysis; and (iii) is positively correlated with individual short-term memory performance. Specifically, we show that successful task performance is associated with consistent phase coupling in these regions across participants, with gamma bursts restricted to specific theta phase ranges corresponding to higher levels of neural excitability. These findings highlight the role of cortico-hippocampal activity in auditory short-term memory and expand our knowledge about the role of cross-frequency coupling as a global biological mechanism for information processing, integration, and memory in the human brain.

## Abstract 2

Cross-frequency coupling in cortico-hippocampal networks enables the maintenance of multiple visuo-spatial items in working memory. This study shows that cross-frequency coupling extends also to auditory information, pointing to a global biological mechanism of cross-frequency coupling for information processing in the human brain.

## Introduction

It is well established that the medial temporal lobe, in particular the hippocampus, is involved in the formation of long-term memories (LTM; [1]). Notably, hippocampal lesions consistently entail LTM deficits (i.e., anterograde amnesia [2]). In contrast, numerous empirical data obtained with a variety of materials, such as words [3], digits [4,5], tones [5], or single-dot locations [4], have led to the hypothesis that hippocampal lesions do not impact working memory (WM) and short-term memory (STM) functions [6,7]. These findings suggest that WM and STM functions rely on distinct processes from LTM (e.g., [8,9]; see also [10,11] for neuroimaging studies).

However, this hypothesis has been challenged by (i) neuropsychological studies reporting that patients with hippocampal lesions experience difficulties in maintaining items in WM or STM [12–14]; and (ii) fMRI [15–17], intracranial EEG [18–21], or single-unit recordings [22,23] in humans reporting persistent, load-dependent, hippocampal activity during WM maintenance of visual information (see also [15] for evidence of hippocampal involvement during auditory STM and [24] for a review about hippocampal activity during general auditory processing).

Hippocampal activity during WM and STM has been originally associated with maintenance-related increase of theta and gamma power [21,25–28]. Interestingly, recent studies went a step further by showing that successful visual memory performance requires the coupling of gamma activity to specific phases of the hippocampal theta (theta-gamma phase amplitude coupling (PAC) [29–32]). Theta-gamma PAC consists in gamma subcycles (local neural activity associated to the processing of each encoded item) that occur at specific theta phase ranges. It has been suggested that theta-gamma PAC plays a critical role in the maintenance of different items in memory and as well as their serial order [31–33]. To date, theta-gamma PAC has been observed in cortico-thalamo-cortical, cortico-cortical, and cortico-hippocampal networks for episodic, working, and long-term memory consolidation in the visual modality [28,34,35]. For the specific case of STM, hippocampal theta-gamma PAC has first been isolated with SEEG in a visual word recognition paradigm in humans: an increased synchronization between the phase of the theta band, and the power changes in the beta and gamma bands were observed when patients successfully remembered previously presented words [36]. Several studies have since confirmed the implication of PAC in STM and WM by showing that the simultaneous maintenance and/or manipulation of multiple visual items in memory is implemented under the form of hippocampal theta-gamma PAC [18,20,37,38].

Overall, previous results suggest that WM or STM maintenance, in which different items must be separately and sequentially maintained over a short period of time, is represented by an ordered activity of cell assemblies implemented under the form of theta-gamma PAC in human cortico-hippocampal networks [31]. However, to date, these studies have mainly focused on visuo-spatial processing, and very little is known about the potential role of theta-gamma PAC in auditory and hippocampal regions during the short-term retention of sequential auditory information. Coupling across cortical oscillations of distinct frequencies in the auditory cortex has been assumed to enable the multiscale sensory analysis of speech (phonemes and syllables [39–41]). However, the direct contribution of auditory-hippocampal cross-frequency coupling for the short-term maintenance of sequential auditory information has not yet been demonstrated. In the present study, we recorded intracranial EEG data while drug-resistant patients with epilepsy performed a delayed matched-to-sample task with tone sequences. If theta-gamma PAC is a predictor of successful memory maintenance, its strength in the auditory and hippocampal regions should (i) be increased during short-term retention of tone sequences (as compared to simple perception); (ii) decode correct and incorrect responses in the STM task using machine learning analysis; and, finally, (iii) be positively correlated with individual auditory STM performance.

## Results

Intracranial EEG recordings were obtained from 16 neurosurgical patients with focal drug-resistant epilepsy. The participants performed an auditory STM task, consisting in the comparison of tone sequences presented in pairs and separated by a silent retention period. In each block of the task, in 50% of the trials, the tone sequences were identical (expected response “same”) and 50% differed by one note (expected response “different”). To manipulate task difficulty, in different conditions, we varied the memory load (3 or 6 to-be-encoded tones, with a tone duration of 250 ms) and the duration of the silent retention period between the to-be-compared sequences (2 s, 4 s, and 8 s; see Table 1 for a detailed description of the conditions and number of participants tested in each condition). Participants also performed a block of listening of the same trials with the instruction to not compare the tone sequences and were simply required to press a button as fast as possible at the end of the last tone of the second sequence (Perception task, 6 tones, 2 s silent period between the tone sequences; see Methods).

**Table 1:** Description of the conditions.

Conditions	Task	Memory load	Retention duration (s)	Number of patients tested
6 tones—short retention	STM	6 tones (total sequence duration 1.5 s)	2	16
6 tones—medium retention	STM	6 tones (total sequence duration 1.5 s)	4	6
6 tones—long retention	STM	6 tones (total sequence duration 1.5 s)	8	16
3 tones—medium retention	STM	3 tones (total sequence duration 0.75 s)	4	6
6 tones -perception task	Do not compare sequences and press 1 key at the end of the second sequence	*6 tones (total sequence duration 1.5 s)*	2	16

STM, short-term memory.

### Accuracy

**Fig 1:**
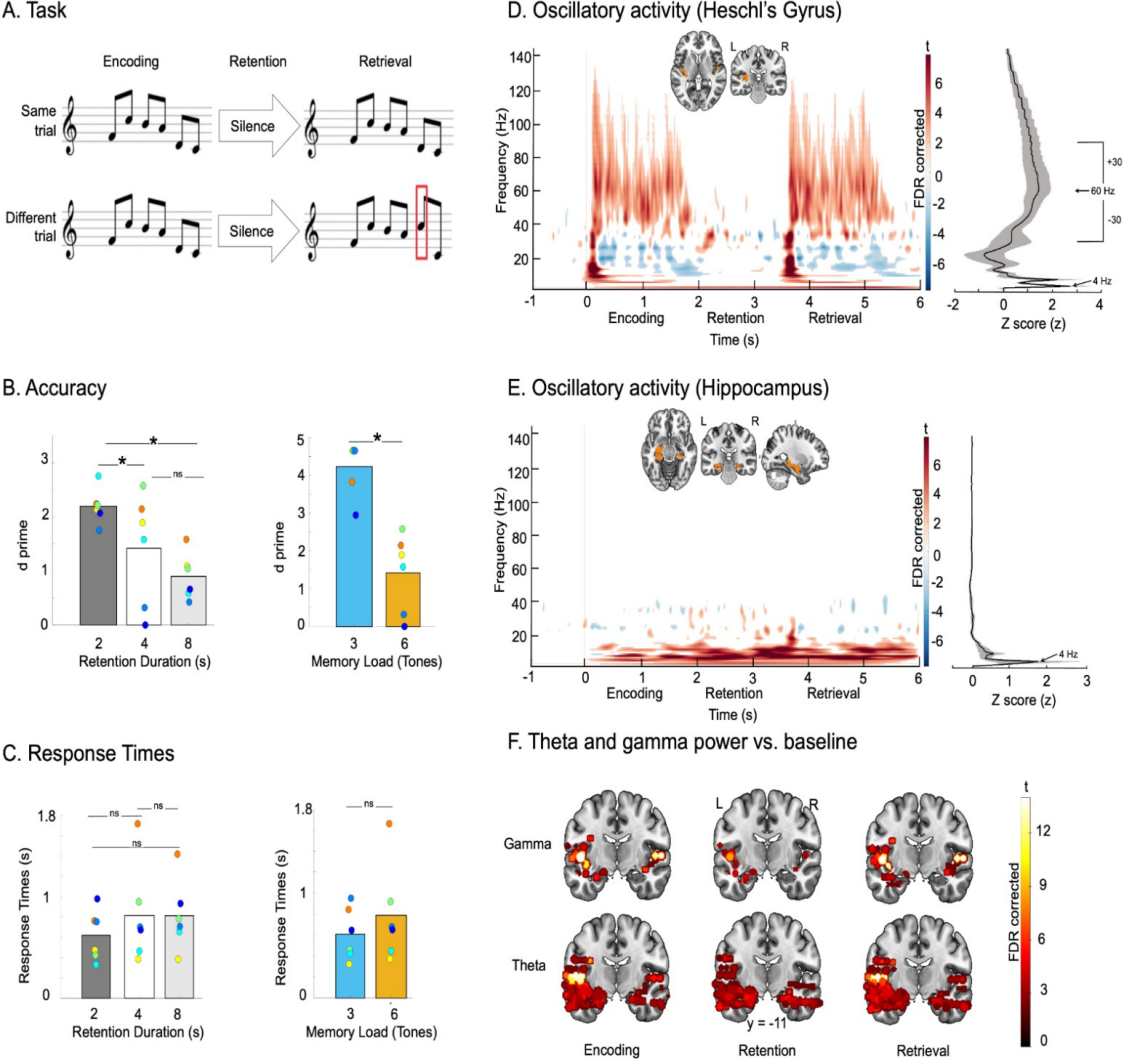
Paradigm, behavioral performance, and brain oscillations. (**A)** Auditory tasks (here with 6-tone sequences, 2 s retention): “Same” trials: After a delay, the first melody was repeated. “Different” trials: One tone was changed in the second melody of the pair in comparison to the first melody (red rectangle). Memory load (3 or 6 tones) and duration of the retention period (2, 4, 8 s) varied in separate blocks. Source data can be found at https://osf.io/m7dta/. (**B)** Accuracy in terms of d prime presented as a function of the duration of the retention period (left panel; *N* = 6) and memory load (right panel; *N* = 6). Colored circles depict participants (one color per participant). Asterisks indicate significance (*p* < 0.05, nonparametric tests; see text for details); NS, nonsignificant. Source data can be found at https://osf.io/m7dta/. (**C)** Response time (s) presented as a function of the duration of the retention period (left panel; *N* = 6) and memory load (right panel; *N* = 6). Colored circles depict participants (one color per participant; same color coding as in Fig 1B). NS, nonsignificant. Source data can be found at https://osf.io/m7dta/. (**D)** Left panel: T-values in the time-frequency domain (*t* test relative to baseline −1,000 to 0 before stimulus onset, FDR corrected in time and frequency domains) of SEEG contacts located in the right and left Heschl’s gyrus (displayed on the single subject T1 in the MNI space provided by SPM12) for a trial time window (−1,000 to 6,000 ms) for the condition 6-tone memory load, 2 s retention period (*n* = 5). Right panel shows the PSD, power spectrum density (zscore) average over a trial time window (0 to 5,000 ms) that was used to define frequency for phase and frequency for amplitude for the PAC analysis. Shaded error bars indicate SEM. Source data can be found at https://osf.io/m7dta/. (**E)** Left panel: T-values in the time-frequency domain (*t* test relative to baseline −1,000 to 0 before stimulus onset, FDR corrected in time and frequency domains) of SEEG contacts located in the right and left hippocampus (displayed on the single subject T1 in the MNI space provided by SPM12) for a trial time window (−1,000 to 6,000 ms) for the condition 6-tone memory load, 2 s retention period (*n* = 14). Right panel shows the PSD, power spectrum density (zscore) average over a trial time window (0 to 5,000 ms) that was used to define frequency for phase for the PAC analysis. Shaded error bars indicate SEM. Source data can be found at https://osf.io/m7dta/. (**F)** SEEG contacts modelled with 4 mm radius spheres (see Methods) in the MRI volume showing a significant increase in oscillatory power (FDR corrected) relative to baseline in theta (4 Hz) and gamma (30–90 Hz) ranges (Hilbert transform averaged over time) during encoding, retention, and retrieval in all memory conditions in all participants (*n* = 16). All results are displayed on the single subject T1 in the MNI space provided by SPM12. Source data can be found at https://osf.io/m7dta/.

Task performance was evaluated using d prime (signal detection theory). To evaluate the impact of the duration of the silent retention period for 6-tone sequences, we performed a nonparametric repeated measures ANOVA (Friedman test) with duration (2 s, 4 s, and 8 s) as a within-participants factor (*n* = 6 participants, note that all participants did not perform all the tasks—see Table 1). The main effect of duration was significant χ^2^ (2) = 7.00, *p* = .03. Post hoc tests performed with Durbin–Conover pairwise comparisons revealed that performance in the 2 s duration condition was significantly better than performance in the 2 other duration conditions (4 s, *p* = 0.004; and 8 s, *p* = .03). Performance in the 4 s and 8 s conditions did not differ significantly (*p* = 0.24, Fig 1B, left panel). To evaluate the impact of memory load on accuracy (3 versus 6 tones with a 4 s silent retention period, *n* = 6 participants), we performed a Wilcoxon rank test revealing, as expected, that performance was increased for the 3-tone condition as compared to the 6-tone condition (W [5] = 21.0, *p* = 0.031; Fig 1B, right panel).

### Response times

The same analyses were performed for response times of correct responses (RTs; Fig 1C) in the same participants (*n* = 6). Nonparametric repeated measures ANOVA (Friedman test) with duration (2 s, 4 s, and 8 s) as a within-participants factor revealed that the main effect of duration of the silent retention period was not significant χ^2^ (2) = 0.33, *p* = .84. In addition, Wilcoxon rank test revealed no significant difference of RTs between the 3-tone condition and the 6-tone condition (4 s silent retention period, W [5] = 4.00, *p* = .21; Fig 1C, right panel).

### Spectral fingerprints of perception and short-term memory of auditory sequences

Fig 1D and 1E show the oscillatory activity (*t* test relative to the baseline −1,000 to 0 ms before stimulus onset, FDR corrected in time and frequency) in the time-frequency domain for SEEG contacts located in the left and right Heschl’s Gyri (according to the AAL3 atlas; see Methods, Fig 1D, 9 SEEG contacts, *n* = 5 participants with one electrode in this area, S1 Table) and bilateral hippocampal and para-hippocampal regions (Fig 1E, 72 SEEG contacts, *n* = 14 participants with one electrode in these areas, S2 Table) for a trial time window for the 6-tone condition, 2 s retention period. Note that the same figures using a logarithmic scale for the frequency axis are presented in S1 Fig. In the auditory cortex, for each tone during the encoding and retrieval periods, transient gamma activity (30 to 90 Hz) was observed. As expected, the encoding of the entire sequence in the auditory cortex was associated with sustained theta oscillations at 4 Hz (tone presentation rate) and at 8 Hz (harmonic; Fig 1D). Moreover, a significant alpha/beta (10 to 20 Hz) desynchronization (relative to baseline) was observed in the auditory cortex during encoding, retrieval, and at the beginning of the retention period (Fig 1D). In the hippocampal and para-hippocampal regions, sustained theta oscillations (4 to 8 Hz) were observed during the entire trial time window (Figs 1E and S1).

We then aimed to evaluate the fluctuations of power relative to baseline in these frequency bands for all SEEG contacts in all participants and all memory conditions. We used Hilbert’s transform (to reduce the dimension of the data) to extract the magnitude of theta (4 Hz) and gamma (30 to 90 Hz) oscillations during encoding, retention, and retrieval periods of the different conditions (averaged in time; see Table 1 for the relevant time periods) for each participant, each SEEG contact, and each trial. A contrast with baseline (FDR corrected) revealed that gamma activity was increased bilaterally in primary and secondary auditory regions and in the hippocampus during encoding retention and retrieval (Fig 1F, top panel; see Supporting information for details and coordinates).

During memory retention, an increase in theta activity was observed in a distributed network including the hippocampal/para-hippocampal regions, inferior frontal gyrus, and several regions of the ventral auditory stream (see Supporting information for details and coordinates; Fig 1F, bottom panel).

To investigate whether these fluctuations of oscillatory power were specific to the memory task, we contrasted memory trials (6 tones, 2 s silent retention delay) with perception trials (6 tones, 2 s silent delay) for each frequency band (theta, gamma) and for all time periods (encoding, retention, retrieval; note that period names apply to the memory task) with nonparametric permutation tests (see Methods and supporting results). To assess significance, we applied a cluster-based approach: We defined SEEG contacts as significant only when they were overlapping for at least 2 participants or 2 SEEG contacts (overlap estimated on an MRI volume where SEEG contacts are represented by spheres with a radius of 4 mm; see Methods). This analysis did not reveal any significant effect for the contrast memory versus perception for each of the periods of the task (encoding, retention, retrieval), all *p*-values > .05 (see S2 Fig plotting theta and gamma power for memory and perception conditions in all SEEG contacts located in regions showing increased theta and gamma power relative to baseline during the retention period).

### Theta-gamma PAC is associated with auditory STM retention

**Fig 2:**
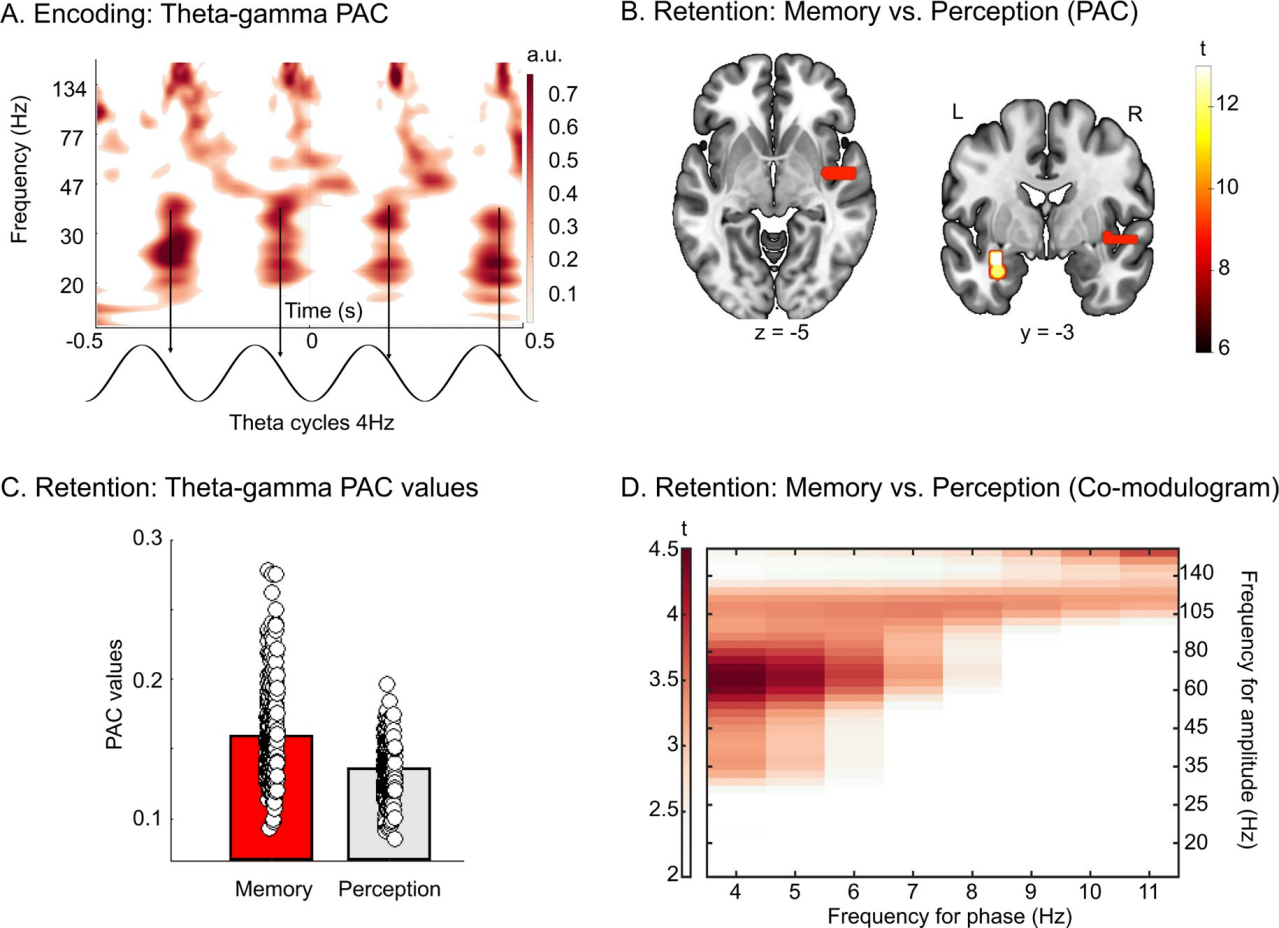
Theta-gamma PAC during encoding and retention. (**A**) Top: Time-frequency plot of mean gamma power modulation time-locked to a 4-Hz (theta) oscillation during encoding in the right and left median belt (*n* = 7). Bottom: Theta (4 Hz) cycles for a 1-s time window. Source data can be found at https://osf.io/m7dta/. (**B**) Memory vs. perception contrast during retention. Top: SEEG contacts (left hippocampus (2 SEEG contacts, *n* = 2) and right auditory areas (15 SEEG contacts, *n* = 1)) showing a significant increase of theta (4 Hz)–gamma (30–90 Hz) PAC strength for memory trials as compared to perception trials during the silent (retention) delay (6 tones, 2 s retention period). All results are displayed on the single subject T1 in the MNI space provided by SPM12. Source data can be found at https://osf.io/m7dta/. (C) Bar plot shows theta-gamma PAC values averaged over trials and participants for memory and perception conditions for the significant SEEG contacts displayed in (**B**). Circles show individual trials. Source data can be found at https://osf.io/m7dta/. (**D**). T-values for the co-modulogram (in SEEG contacts identified in B) for memory versus perception contrast (*p* < .05, FDR corrected). Source data can be found at https://osf.io/m7dta/.

Notwithstanding the fact that no effect was observed for the memory versus perception contrast on theta and gamma power, we investigated whether theta-gamma PAC during memory retention could rather be a more specific marker of STM retention. For all PAC analyses, we adopted the following strategy: All analyses, except the memory versus perception contrast (see Table 1 and Fig 2), were done within subject, for all participants, using all data of the memory conditions. We then report only the significant SEEG contacts that were overlapping between participants or between electrodes using a cluster procedure (see below and Methods). As expected, during encoding, clear transient gamma oscillations were nested in the theta cycle (Fig 2A for illustration) in the auditory cortex (Heschl’s gyrus, 9 SEEG contacts, *n* = 5 participants, S1 Table). To investigate whether this mechanism played a functional role during retention, we contrasted the theta-gamma PAC strength values of memory trials (6 tones, 2 s retention) with the theta-gamma PAC strength values of perception trials (6 tones, 2 s retention) during the retention period (permutation testing, 10,000 permutations), for each participant and each of their SEEG contacts (Fig 2B). After computing this analysis for each participant, we used the same cluster-based approach as for the analysis of oscillatory power (see Methods). This analysis revealed a clear increase in theta-gamma PAC in the left hippocampus (2 SEEG contacts, *n* = 2) and right auditory regions (15 SEEG contacts, *n* = 1) in the memory condition compared to the perception condition (Fig 2B and 2C, all ps < 0.001; see S3 Table for coordinates).

However, one can question whether this coupling was specific to theta and gamma oscillations as theta-beta, alpha-gamma, and alpha-beta PAC have previously been reported during working memory [42]. To test whether this effect was specific to the phase of the theta and the amplitude of the gamma oscillations, we computed the same analysis in the SEEG contacts showing significant PAC increase in the memory versus perception contrast (displayed Fig 2B; see S3 Table for details and coordinates), but using multiple low frequencies as frequency for phase (4 to 11 Hz, i.e., theta to alpha) and multiple high frequencies as frequency for amplitude (15 to 140 Hz, i.e., beta to high gamma; see Fig 2D). Interestingly, the memory versus perception contrast performed on these co-modulograms (*p* < .05, FDR corrected) revealed that the maximum increase in PAC strength for memory trials as compared to perception trials was observed between theta (4 to 6 Hz) as frequency for phase and gamma as frequency for amplitude (35 to 105 Hz). Note that we performed the same analysis in all SEEG contacts located in regions showing increased theta and gamma power relative to baseline during retention (Fig 1F, middle panel, coordinates in the Supporting information). This analysis revealed no significant difference of PAC strength between memory and perception trials after FDR correction (see S3 Fig for illustration of the difference of PAC strength values between memory and perception trials)

### Theta-gamma PAC in fronto-temporal areas and hippocampus decodes correct and incorrect memory trials and correlates with auditory STM performance

We then investigated whether the strength of theta-gamma PAC during memory retention can decode correct and incorrect memory trials and predict STM performance. To do so, we used the SEEG data and the behavioral data of all memory conditions for each participant. We first used a support vector machine (SVM) classifier with 3-fold cross-validation to classify correct and incorrect trials in all memory conditions, using only PAC strength in each SEEG contact as input features (see Methods). This approach was implemented for each participant: The model is trained only on data from 2/3 of the trials to predict whether a trial is correct or incorrect in the remaining 1/3 of the trials. The procedure is repeated 3 times, and the summary of the SVM’s performance (average of all models) reflects, for each participant, the degree to which correct and incorrect STM trials can be discriminated based on PAC strength. As all participants had more correct than incorrect trials for all memory conditions, we made a random selection of the correct trials (to match the number of incorrect trials for each condition) to train and test the classifier. Then, we repeated this analysis 100 times with 100 different random selection of correct trials for each participant. SVM’s performance was evaluated using the output of the 100 models (accuracy minus chance) for each participant.

**Fig 3:**
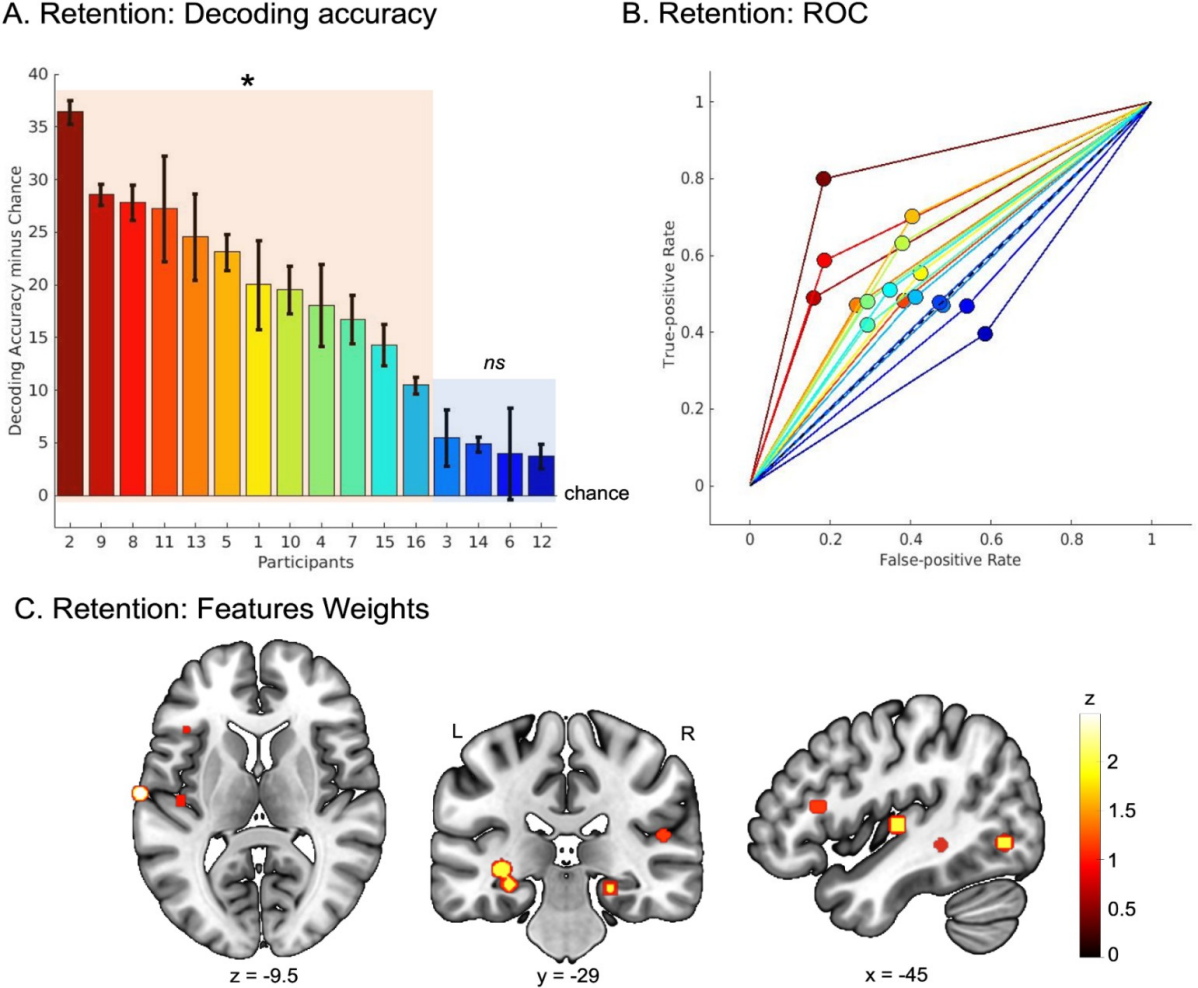
PAC as markers of correct vs. incorrect memory retention identified with machine learning. (**A**) SVM decoding accuracy (accuracy minus chance—chance level: 0%) for a 2-class decoding analysis of PAC strength and SEEG contacts as features (correct vs. incorrect memory retention in all memory conditions). The colored bars represent accuracy minus chance for each participant (sorted as a function of accuracy with a jet colormap). Orange shaded rectangle overlaps with participants showing decoding accuracy significantly above chance. Blue shaded rectangle overlaps with participants with decoding accuracy not significantly different from chance. Asterisk: significant, ns: nonsignificant. Source data can be found at https://osf.io/m7dta/ (**B**) ROC for each participant (same color code as in A). Black dashed line represents the chance level. Source data can be found at https://osf.io/m7dta/. (**C**) Normalized feature weights showing features (SEEG contacts) with the largest influence (z-score) for each participant with significant decoding accuracy. Source data can be found at https://osf.io/m7dta/. PAC, phase amplitude coupling; ROC, receiver operating characteristic curve; SVM, support vector machine.

The models significantly classified correct and incorrect memory trials above chance in 12/16 participants (all ps < .03 as measured by a Wilcoxon rank test; Fig 3A; ROC curves for each participant are presented in Fig 3B). We then aimed to define the SEEG features (i.e., SEEG contacts) the models relied upon to discriminate correct and incorrect STM trials. For each participant with significant above chance decoding accuracy, we extracted the feature weights to estimate their relative importance (z-scored, normalized across features for each participant) in the classification. We then extracted the SEEG contact showing the maximum zscore value (i.e., contributing more to the classification) for each participant and represented it on a MRI volume (Fig 3C). This analysis revealed that the right and left hippocampus, the right IFG, the right and left primary auditory cortices, the left STS, and the left ITG (see S4 Table for details) were the brain regions where PAC strength allowed to classify correct and incorrect memory trials.

It is relevant to note, however, that this analysis does not allow to infer whether PAC strength in the identified brain regions was associated to good or poor performance. Indeed, the features weights shown in Fig 3C can be used only to infer that PAC strength in these given SEEG contacts can decode correct and incorrect memory trials.

**Fig 4:**
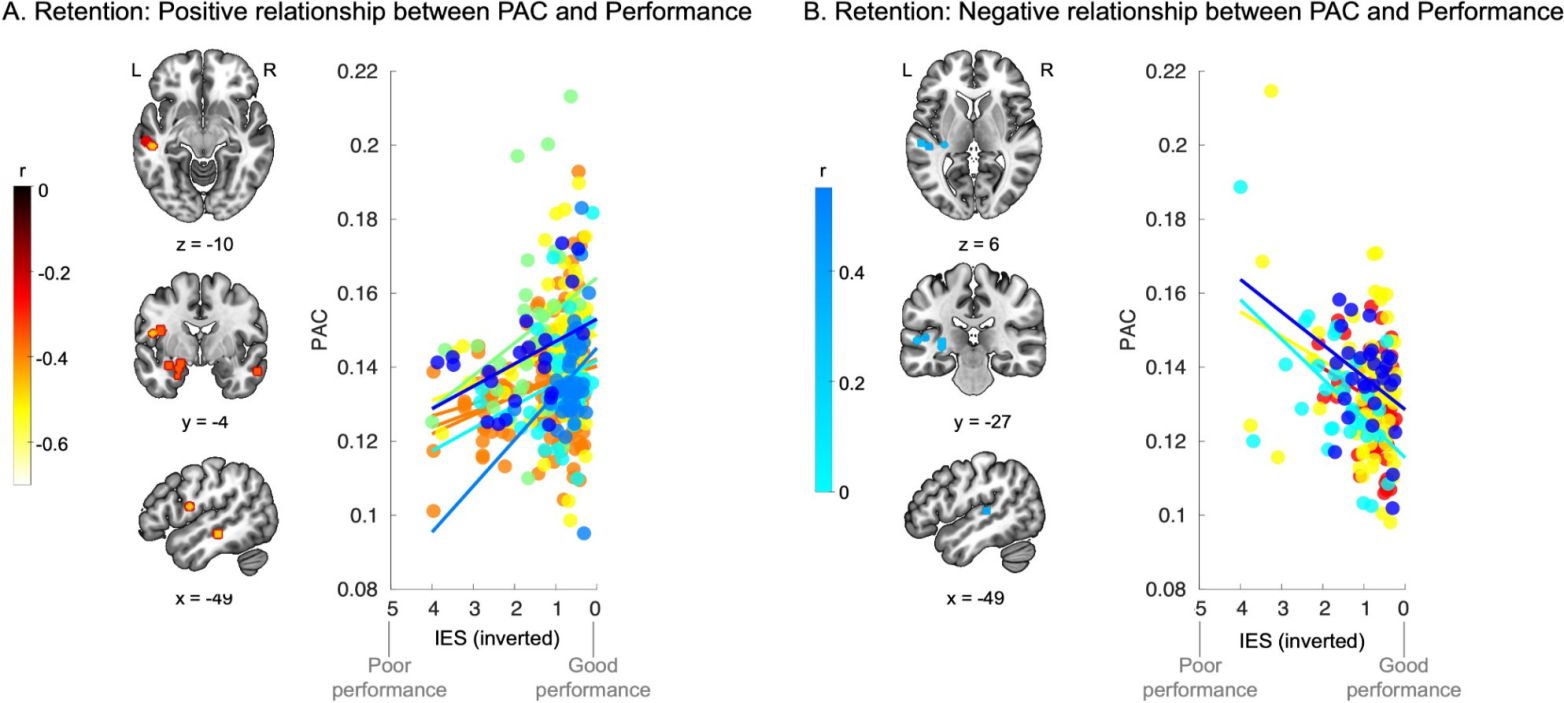
Theta-gamma PAC in the hippocampus and ventral auditory stream correlates with behavior. (**A**) Left panel: SEEG contacts showing a positive correlational relationship between theta-gamma PAC and performance (negative correlation with IES). Results are displayed on the single subject T1 in the MNI space provided by SPM12. Right panel: Scatter plot of IES (note that the scale is inverted for clarity: 5 corresponding to poor performance and 0 corresponding to good performance) against theta-gamma PAC strength for each significant SEEG contact. Each color depicts a different participant (*N* = 6). Source data can be found at https://osf.io/m7dta/. (**B**) Left panel: SEEG contacts showing a negative correlational relationship between theta-gamma PAC and performance (positive correlation with IES). Results are displayed on the single subject T1 in the MNI space provided by SPM12. Right panel: Scatter plot of IES (note that the scale is inverted for clarity: 5 corresponding to poor performance and 0 corresponding to good performance) against theta-gamma PAC strength for each significant SEEG contact. Colors show the different participant (*N* = 4). Source data can be found at https://osf.io/m7dta/. IES, inverse efficiency score; PAC, phase amplitude coupling.

We thus investigated whether theta-gamma PAC during memory retention can be correlated to STM performance. To do so, we used the SEEG data and the behavioral data of all memory conditions for each participant. This allowed us to benefit from the variability in behavioral performance associated with the manipulation of the memory load and of the duration of the retention period. As a significant effect of condition emerged for the accuracy data (Fig 1B), but not for the RT data (Fig 1C), we computed for each trial the inverse efficiency score (IES; correct RT at the single trial scale/percent correct in the corresponding condition; see [43] and Methods). This behavioral metric increased the variability of behavioral scores between memory conditions with a low score representing a rapid RT and a high percentage of correctness. We then performed a Pearson’s correlation between IES and PAC strength values for each SEEG contact and each participant (across all conditions). This analysis revealed, after cluster correction, that theta-gamma PAC values in the left hippocampus (4 SEEG contacts, *n* = 2), left superior temporal sulcus (STS; 2 SEEG contacts, *n* = 2), right inferior temporal gyrus (ITG; 2 SEEG contacts, *n* = 2), and left inferior frontal gyrus/insula (IFG; 2 SEEG contacts, *n* = 2) had a positive correlational relationship with performance (i.e., negatively correlated with the IES; Fig 4A and see S5 Table). Moreover, this analysis also revealed that theta-gamma PAC in the left Heschl’s gyrus (4 SEEG contacts, *n* = 4) had a negative relationship with performance (positively correlated with the IES; Fig 4B and S6 Table). Note that we performed the same analysis only with the conditions that were performed by all 16 participants (see Table 1) and obtained similar results (see S4 Fig).

### Coupling phase is consistent across participants and trials

**Fig 5:**
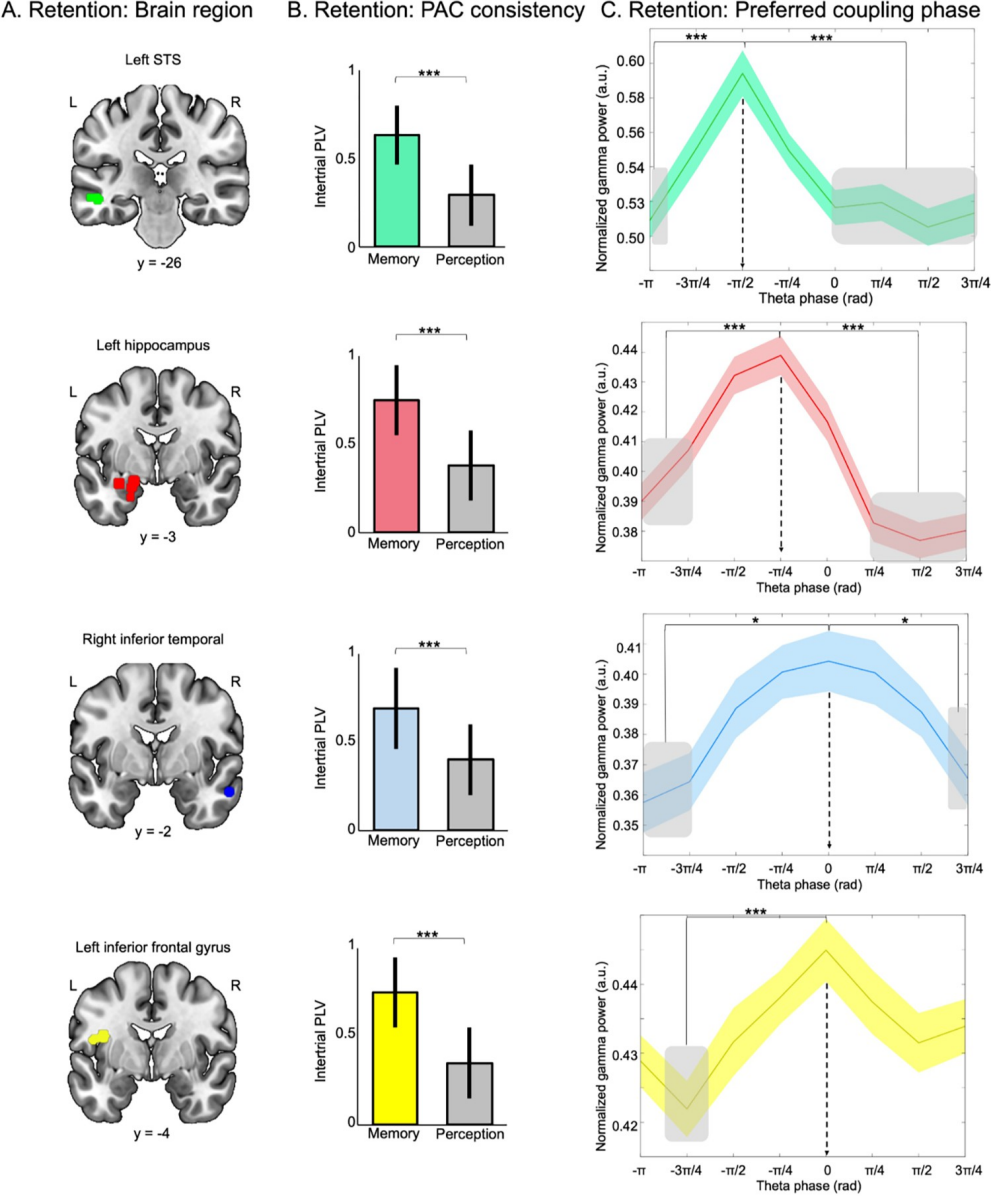
Theta gamma PAC is consistent across trials and participants. (**A**) SEEG contacts identified in Fig 4A and grouped as a function of their location according to the AAL Atlas: green, left STS; red, left hippocampus; blue, right ITG; yellow, left IFG/insula. Regions are displayed on the single subject T1 in the MNI space provided by SPM12. Source data can be found at https://osf.io/m7dta/. (**B**) PAC intertrial phase consistency computed for each region. Bar plot shows intertrial phase locking values across participants and SEEG contacts for memory trials (correct responses, colored as a function of the regions) and perception trials in the same region. Error bars indicate SEM. Asterisk indicates significance. Source data can be found at https://osf.io/m7dta/. (**C**) Preferred coupling phase: gamma power presented as a function of theta phase bins for each region. Shading represents the standard deviation across trials and participants. Asterisks (*** *p* < .001; * *p* < .05) and grey shading indicate significance. Note that for clarity, we show only the results for the post hoc tests performed for the peak of gamma power for each region. Detailed post hoc statistics are reported in S7–S10 Tables. Source data can be found at https://osf.io/m7dta/. IFG, inferior frontal gyrus; ITG, inferior temporal gyrus; PAC, phase amplitude coupling; STS, superior temporal sulcus.

The analyses presented in Figs 2 to 4 evaluated PAC strength for each participant (coupling consistent across trials, within participant). However, these analyses do not guarantee that the coupling occurred at the same phase for all participants: Different participants could show a preferred coupling at different phases of the theta oscillations. To investigate this question, we further evaluated whether gamma bursts were consistently restricted to specific phase ranges of the theta oscillations across participants in regions identified in Fig 4A (using data of all conditions available for the participants showing significant effects in Fig 4A). We first computed the theta-gamma phase consistency across trials, for the SEEG contacts where the PAC strength was correlated with behavioral performance (see Fig 4A and S5 Table). For each trial, and each SEEG contact, we extracted the magnitude of gamma oscillations (30 to 90 Hz) as a function of the phase of the theta oscillation (4 Hz) (average over the entire retention period, theta phase divided into 8 bins; see Methods). In both memory (correct trials) and perception trials separately, we computed the intertrial phase locking value (PLV) as a measure of intertrial phase consistency of the coupling. Then, this metric was contrasted between memory and perception trials (Wilcoxon rank test) for each region (grouping SEEG contacts as a function of their location in the AAL atlas; Fig 5A). As expected, this analysis revealed greater consistency in theta-gamma PAC for memory as compared to perception trials for all regions (all *p*-values < .0001; Fig 5B).

Finally, we aimed to identify whether a specific coupling phase range between the phase of the theta oscillations and the amplitude of gamma oscillations can be identified in these regions across trials and participants. To do so, we used linear mixed models (LMM) and modeled the variability between participants by defining by-participant random intercepts. This analysis was done for each region with theta phase bin as fixed factors and participants as a random factor (using data of all memory conditions available for the participants showing significant effects in Fig 4A). For all regions, we observed a main effect of theta phase (all χ2 (7) > 18.7; all *ps* < .01) on the gamma power. Post hoc Tukey analysis revealed increased gamma power between −π/2 and 0 of the theta cycle as compared to other bins in all regions (Fig 5C, see S7–S10 Tables for detailed statistics).

## Discussion

Using intracranial electrophysiological recordings in humans, we showed that (i) the strength of theta-gamma PAC in temporal regions and hippocampus was increased during the short-term retention of auditory sequences as compared to simple perception; (ii) the strength of theta-gamma PAC in STS, ITG, IFG, and hippocampus decode correct and incorrect memory trials as evaluated with machine learning; (iii) the strength of theta-gamma PAC in these regions was positively correlated with individual STM performance; and, finally, that (iv) the coupling phase was highly consistent in these regions across individual participants to enable successful memory performance (high-frequency oscillations consistently restricted to specific phase ranges of the theta oscillations). The implications of these findings are discussed below.

### Increasing memory load and duration of the silent retention period decrease performance

In line with previous studies, the present behavioral findings indicated that participants’ STM abilities (as also observed for other materials, such as verbal or visuo-spatial) decreased with increasing duration of the silent retention period [44] and increasing memory load ([45]; see Fig 1B). In the present study, we used these manipulations to increase the variability in task difficulty (and, consequently, modulate participants’ behavioral performance) across conditions. By combining information from accuracy and response times, we extracted a behavioral measure for each trial (IES; see methods and [43]) that we used to investigate the link between PAC strength values and behavior for each participant.

### Brain networks of auditory perception and short-term memory

Time-frequency analyses revealed that transient gamma activity was evoked by each tone of the sequence in the auditory cortex, secondary auditory regions, hippocampus, and several areas of the ventral pathway during the encoding and retrieval periods of the STM task and the equivalent periods of the perception task (see Fig 1C and 1D). It is well established that gamma oscillations are marking bottom-up and local (intraregional) processes during both passive and active sensory integration [46,47]. Observing such transient bursts after each tone of the to-be-encoded sequence can thus be considered as a marker of the integration of tones’ features by the sensory system (bottom-up).

In addition, sustained theta oscillations were observed in distributed regions of the ventral pathway, including STS, STG, IFG, and hippocampus (see Supporting information) during encoding, retention, and retrieval. Theta oscillations (4 to 8 Hz) are typically considered as markers of attention, arousal, or memory during demanding cognitive tasks [48–50]. Notably, theta oscillations are known to play a key role in ordering items that are presented sequentially in STM or WM [51]. Moreover, theta oscillations have been associated to long-range communication between distant brain regions during memory maintenance [49,50,52–54]. In the present study, an increase relative to baseline in theta power was observed in the hippocampus, inferior frontal regions, and secondary auditory regions, a brain network that has been consistently reported as being recruited during auditory STM tasks [15,55–57] (Fig 1F).

However, during all phases of the task (referred to as encoding, retention, and retrieval periods for the memory task and their equivalent for the perception task), we did not observe any significant differences of gamma and theta magnitude between memory and perception trials. This result contrasts with the studies reported above [49,50,52–54]. A possible interpretation would be that the participants have been carrying out a form of WM during the perception task (always performed after the memory condition; see Methods) even if they were not instructed to do so. An alternative interpretation would be that the fluctuations in oscillatory magnitude in the theta and gamma frequency ranges extracted in the present study were not specific to memory and might rather be associated with the perception of the sequence and attention towards the auditory input (note that even in the perception task, participants had to pay attention to the sound sequences to push a button at the end of S2).We thus aimed to define whether more fine-grained oscillatory markers related to memory retention can be identified with the investigation of theta-gamma PAC.

### Theta-gamma PAC in auditory and hippocampal regions is associated to auditory short-term memory retention

During encoding, we observed that gamma oscillations were nested in the theta cycle in the auditory cortex (see Fig 2A for illustration). This effect was expected as each tone of the sequence induced a time-locked (or evoked) increase in gamma power, and the phase of the theta oscillation was entrained by the tone presentation rate (4 Hz; see [49,54] for basic principles of sensory entrainment). We then investigated whether this statistical dependency between the phase of theta oscillations and the amplitude of gamma oscillations was still present during the retention period, a time window for which no stimuli were presented. More specifically, we investigated whether PAC signals were increased during memory retention as compared to perception. In the left hippocampus and right temporal regions, the strength of theta-gamma PAC was indeed significantly higher during the retention delay in the memory condition compared to the perception condition (see Fig 2B and S3 Table). It is relevant to note that this effect was observed in a limited number of SEEG contacts and participants. This is related to the cluster correction procedure we have used that keep only SEEG contacts that overlap between participants or contacts. One possible interpretation is that PAC during memory retention could result from sustained PAC signals that originally emerged during encoding (see Fig 2A; PAC coming from bottom-up entrainment at 4 Hz). It can thus be argued that the significant effect observed between memory retention and perception could result from attentional differences for memory and perception trials during encoding (participants paying more attention during memory than perception trials). However, one can argue that attentional effects could not only be observed in PAC measures but could also affect theta and gamma magnitude [58]. As the contrast between memory trials and perception trials for theta and gamma magnitude was not significant in the present study, we propose that these PAC effects were specific to memory.

These results thus suggest a role of the hippocampus in auditory STM. This is in line with several neuroimaging studies in the visual modality [16,18,19,38] and also with recent single-unit recording studies in humans reporting increased neural firing in the hippocampus during the maintenance of visual representations [22,23,59]. For auditory STM, hippocampal involvement has, however, been less frequently described in previous research. Using an auditory STM task during fMRI recordings, Kumar and colleagues [15] have shown sustained activity in both ventral and dorsal parts of the hippocampus during an auditory STM task. Here, we observed activity mainly in its ventral part (y = −4), a finding fitting well with the fact that the anterior portion of the hippocampus is anatomically and functionally connected to auditory areas [60,61]. Interestingly, Kumar and colleagues [15] also reported that the pattern of fMRI activity in hippocampal areas allows the decoding of the different sounds maintained in memory. Our present study goes beyond these findings by identifying the neurophysiological mechanism by which the hippocampus supports retention of auditory information in memory.

Indeed, here we showed that theta-gamma PAC in the hippocampus and temporal regions (STS, ITG) decodes correct and incorrect memory trials (Fig 3A and S4 Table) and was positively correlated with behavioral performance (negative correlation with IES; Fig 4A and 4B and S5 Table). This finding is well aligned with previous research showing that hippocampal theta-gamma PAC plays a functional role during memory retention for visual material [18,20,37,38]. In the present study, we show that the temporal and hippocampal regions implement the same electrophysiological mechanism to allow for the maintenance of sequential auditory information, a finding that has, to our knowledge, never been reported before. This finding is also well aligned with a recent study showing cortico-hippocampal interplay in the theta range during both encoding and retention of a STM task with visually presented words [62]. Taken together, our results suggest a clear role of theta-gamma PAC in the temporal and hippocampal regions during auditory STM in the human brain.

In addition to auditory and hippocampal regions, we observed that theta-gamma PAC strength in the left IFG decodes correct and incorrect memory trials (Fig 3A and S4 Table) and was positively correlated with behavioral performance (negative correlation with IES; Fig 4A and S5 Table). This is in line with the well-established role of the IFG in STM maintenance in humans [15,50,55–57,63–69]. Interestingly, we also observed that theta-gamma PAC in Heschl’s gyrus during memory retention was negatively correlated with behavioral performance (positive correlational relationship with IES; Fig 4B). This result suggests that to perform successfully the STM task, PAC signals need to reach higher-level regions, namely, STS, ITG, hippocampus, and inferior frontal regions, to allow for efficient maintenance of the information. This hypothesis receives support in a recent study showing that theta and gamma activity in the human hippocampus is associated with successful recall when extrahippocampal activation patterns shifted from perceptual toward mnemonic representations. This study also suggests that recurrent hippocampal–cortical interactions are then implemented to support memory processing [70].

From a more global perspective, our results are in agreement with the theta-gamma neural code hypothesis developed by Lisman and Jensen [31], proposing that cross-frequency signaling in cortico-hippocampal networks is a sophisticated mechanism implanted by the brain to hold sequentially organized information in memory [20,25,31]. This hypothesis assumes that representations of individual encoded items (via high-frequency oscillations) do not occur during the entire cycle of low-frequency oscillations. Instead, these high-frequency oscillations are thought to be restricted to specific phase ranges of the slow oscillation that correspond to higher levels of neural excitability [20,31,71]. To test the validity of this model, we investigated for each region whether the gamma bursts in the present data were consistently restricted to a specific phase range of the theta oscillations across trials and participants.

### Consistent phase coupling across participants during successful memory performance

We extracted the PAC consistency across trials and participants in the brain regions where PAC strength was positively predicting behavioural performance (see Fig 5A and S5 Table). Intertrial-phase locking analysis on these signals revealed greater consistency in theta-gamma PAC for memory trials than for perception trials in all regions (Fig 5B). We then aimed to identify whether a preferred coupling phase range could be identified. We observed that, for correct memory trials, the gamma bursts were occurring consistently at a specific phase range of the theta cycle in the left STS, right ITG, left IFG, and the left hippocampus (see Fig 5C and S7–S10 Tables). This preferred phase is of interest because it suggests that similar mechanisms are implemented in this network across trials and participants. Interestingly, the gamma burst occurred from the trough of the theta cycle to its peak. As shown in earlier research, the phase of theta oscillation reflects rhythmic fluctuations of neural excitability [72]. Such cycles, occurring several times per second, represent fluctuations between (high-excitability) phases during which relevant information is amplified and (low-excitability) phases during which information is suppressed. Here, we observed high coupling consistency between −π/2 and 0 of the theta cycle, a phase range corresponding to a high-excitability period of the oscillation where information processing can be amplified [25,31,72]. Observing this effect only for correct memory trials is another important cue suggesting that fronto-auditory-hippocampal theta-gamma PAC allows successful integration and the retention of sequential auditory information in STM.

Overall, our study provides new information about the neurophysiological mechanisms by which the fronto-temporal-hippocampal network encodes and maintains sequential auditory information. The findings provide crucial insights into the networks and brain dynamics involved in this fundamental process in the auditory modality.

## Methods

### Participants

Intracranial recordings were obtained from 16 neurosurgical patients with drug-resistant focal epilepsy (8 females and 8 males, mean age: 32.6 +/− 8.73 years) at the Epilepsy Department of the Grenoble Neurological Hospital (Grenoble, France) and the Epilepsy Department of Lyon Neurological Hospital (Lyon, France). All patients were stereotactically implanted with multilead EEG depth electrodes. Data from all electrodes exhibiting pathological waveforms were discarded from the present study. All participants provided written informed consent, and the experimental procedures were approved by the appropriate regional ethics committee (CPP Sud-Est V, 2009-A00239-48). The study has been conducted according to the principles expressed in the Declaration of Helsinki.

### Task and conditions

The participants were asked to perform an auditory STM task, consisting in the comparison of tone sequences presented in pairs and separated by a silent retention period. Participants also performed a block of passive listening of these trials in which they were required to ignore the content of tone sequences and press a button as fast as possible at the end of S2. To manipulate task difficulty (only for the memory task), in different blocks, we varied the memory load (3 or 6 to-be-encoded items) as well as the duration of the silent retention period between the to-be-compared sequences (2 s, 4 s, and 8 s; see Table 1 for a detailed description of the conditions). All tone sequences were composed of 250-ms-long piano tones presented sequentially without interstimulus interval. The 2 sequences could be either the same or different (50% of each trial type). For “different” trials, the second sequence differed by a single tone altering the melodic contour (Fig 1A). For the 6-tone melodies, 120 different tone sequences were created using 8 piano tones differing in pitch height (Cubase software, melodies from [55]); all used tones belonged to the key of C Major (C3, D3, E3, F3, G3, A3, B3, C4). For the 3-tone sequences, 60 different tone sequences were created using the same pool of piano tones (material from [55,56]).

### Procedure

Presentation software (Neurobehavioral Systems, Albany, CA, USA) was used for the delivery of the experimental protocol to present the auditory stimuli and to register button presses. For each trial, participants listened binaurally (presented with headphone at a comfortable listening level) to the first 3- or 6-tone sequence with a total respective duration of 750 or 1,500 ms (encoding, S1), followed by a silent retention period (2, 4, or 8 s), and then the second sequence (retrieval, S2, 750 or 1,500 ms duration). Conditions were counterbalanced across participants. Participants were informed of the block order and were asked to indicate their answers by pressing one of 2 keys with their right hand after the end of S2. Their responses were recorded during the first 2 s of the intertrial interval, whose random duration was comprised between 2.5 and 3 s. No feedback was given during the experiment. Each block of the task included 30 trials (15 “same” trials and 15 “different” trials for each condition). Within each block, the trials were presented in a pseudorandomized order; the same trial type (i.e., “same” or “different”) could not be repeated more than 3 times in a row. Before the first session, participants performed a set of 10 practice trials (with melodies not used in the main experiment).

### Analysis of behavioral data

Task performance was measured with d prime (Signal Detection Theory). RTs were measured from the end of S2. Behavioral data were analyzed with nonparametric repeated measures ANOVA (Friedman) and Wilcoxon rank test (see Results). The IES was calculated for each trial. IES is computed by normalizing, at the single trial scale, the participant RT by their respective percentage of correct responses in each condition. As compared to RTs, this behavioural metric increases the variability of behavioural scores with a low score representing a short RT and a high percentage of correctness [43]. Correlation analysis between performance at the single trial level and brain data (PAC values; see below) were performed using IES.

### Localization of depth electrodes

In each patient’s brain, 10 to 16 semirigid, multilead electrodes were stereotactically implanted. The SEEG electrodes had a diameter of 0.8 mm and, depending on the target structure, consist of 10 to 15 contact leads 2.0 mm wide and 1.5 mm apart (DIXI Medical Instruments). All participants underwent two 3D anatomical MPRAGE T1-weighted MRI scan on a 1.5T Siemens Sonata scanner or on a 3T Siemens Trio (Siemens AG, Erlangen, Germany) before implantation and just after the SEEG implantation. The anatomical volume consisted of 160 sagittal slices with 1 mm3 voxel, covering the whole brain. The scalp and cortical surfaces were extracted from the T1-weighted anatomical MRI. All electrode contacts were identified on the post-implantation MRI showing the electrodes and coregistered on a pre-implantation MRI (ImaGIN toolbox; https://f-tract.eu/software/imagin/). MNI coordinates were computed using the SPM (http://www.fil.ion.ucl.ac.uk/spm/) toolbox. In addition to MNI coordinates, we computed the localization of the SEEG contacts in the AAL3 atlas [73].

### Intracranial recordings

Intracranial recordings were conducted using a video-SEEG monitoring system (Micromed), which allowed the simultaneous data recording from 128 depth EEG electrode sites (identical acquisition system and acquisition parameters in the 2 recording sites). The data were bandpass filtered online from 0.1 to 200 Hz and sampled at 512 Hz for all patients. At the time of acquisition, data were recorded using a reference electrode located in white matter, and each electrode trace was subsequently re-referenced to its immediate neighbour (bipolar derivations). This bipolar montage has several advantages over common referencing. It helps eliminating signal artifacts common to adjacent electrode contacts (such as the 50 Hz mains artifact or distant physiological artifacts) and achieves a high local specificity by cancelling out effects of distant sources that spread equally to both adjacent sites through volume conduction. The spatial resolution achieved by the bipolar SEEG is estimated to be on the order of 3 mm [74].

### Preprocessing

SEEG data were preprocessed and visually checked to reject contacts contaminated by pathological epileptic activity or environmental artifacts. Powerline contamination of the raw data (main 50 Hz, harmonics 100 and 150 Hz) was reduced using notch filtering. Then, data were epoched to create trials with a window of 1,000 ms before the onset of S1 and 500 ms after the end of the last stimulus of the S2 sequence. SEEG contacts showing signal values exceeding 1,500 μV during the trial time window were excluded from the analysis: As a result, between 17 and 30 trials were kept for each participant and condition.

### Time-frequency analysis in Heschl’s gyrus and hippocampus

We first performed time-frequency Morlet analysis for the SEEG contacts located in the right and left Heschl’s gyrus and bilateral hippocampus (according to the AAL atlas). This analysis was done to define the frequency bands of interest for the whole brain Hilbert’s analysis and to define the frequency for phase and frequency for amplitude for the PAC analysis. Time-frequency Morlet analysis was computed based on a wavelet transform of the signals [75]. The wavelet family was defined by (f0 /sf) = 7 with f0 ranging from 1 to 150 Hz in 1 Hz steps. The time-frequency wavelet transform was applied to each SEEG contact, each trial, and then power was averaged across trials, resulting in an estimate of oscillatory power at each time sample and each frequency bin between 1 and 150 Hz. Note that both evoked and induced activity were estimated. We then performed a normalization (z-scoring) with −1,000 to 0 ms preceding the presentation of the S1 sequence as baseline. Time-frequency plots of SEEG contacts were regrouped in left and right Heschl’s gyrus and bilateral hippocampus across participants using the AAL3 brain atlas. By doing so, we were able to investigate the data of several participants on one time-frequency map per area. Normalized and averaged time-frequency maps of the auditory cortex and hippocampus were used to define the frequency for phase and frequency for amplitude for the PAC analysis (see below). Frequency for amplitude was defined from 30 Hz to 90 Hz as it matched with the amplitude of time-frequency maps gamma bursts in the auditory cortex (see also [18] for similar parameters). Frequency for phase was defined at 4 Hz because sustained theta power at 4 Hz was observed in the auditory cortex during encoding (Fig 1D), and this frequency matched the frequency of presentation of the stimuli.

### Hilbert transform

Once the frequency bands of interest were defined, we aimed to investigate if fluctuation of theta and gamma power were associated to memory processes (as compared to perception). In order to perform this analysis at the whole brain level and to reduce the dimension of the data, we computed for each participant the Hilbert transform for correct trials for each period of the STM task (encoding, retention, and retrieval, average in time for each time period; see Table 1) and the corresponding periods of the perception task. We extracted the magnitude of theta activity at 4 Hz and gamma activity between 30 to 90 Hz for each trial for each SEEG contact. These data were then used to contrast brain activity in the memory conditions and baseline and to contrast brain activity in the memory and perception conditions using permutation tests as implemented in MATLAB. Contrasts with baseline were corrected for multiple comparison using FDR corrections. Memory versus perception contrast were corrected with a cluster procedure (see below).

### Phase amplitude coupling

Theta-gamma PAC was computed using the method developed by [76]. Frequency for phase and frequencies for amplitudes were defined by a power spectrum density analysis on SEEG contacts located in the auditory cortex and in the hippocampus and computed over the total duration of a trial time window (0 to 5.5 s for the 6 tones, 2 s memory condition as this condition was performed by all 16 participants). Frequency for phase was selected as the frequency showing the highest peak in the theta band (4 to 8 Hz) in the auditory cortex and hippocampus (see Fig 1D and 1E) and frequency for amplitude was defined as a 60-Hz-width frequency band centered on the highest peak in the gamma band (peak at 60 Hz ± 30 Hz resulting in a band between 30 and 90 Hz) in the auditory cortex. Based on these results (see Fig 1D and 1E), we used 4 Hz as the frequency for phase (frequency of presentation of stimuli) and 30 to 90 Hz as the frequency for amplitude for the PAC analyses. As no high gamma peak emerged in this PSD analysis, we did not investigate PAC for frequencies above 90 Hz.

### 3D representation and cluster procedure

For all PAC analyses and Hilbert data, significant SEEG contacts were plotted on a MNI MRI volume using marsbar and SPM functions [77]. To do so, we extracted the MNI coordinate of each SEEG contact and represent the oscillatory magnitude and PAC values on spheres of 4 mm radius in the MRI volume. PAC plots were corrected with a cluster approach: by considering as significant only the contacts that were overlapping across at least 2 participants or 2 SEEG contacts in the MRI volume.

### Multivariate analyses

Multivariate analyses were performed using MATLAB and SVM implementation (https://www.mathworks.com/help/stats/fitcecoc.html). A linear classifier was chosen as SEEG data contains many more features than examples, and classification of such data is generally susceptible to overfitting. One way of alleviating the danger of overfitting is to choose a simple function (such as a linear function) for classification, where each feature affects the prediction solely via its weight and without interaction with other features (rather than more complex classifiers, such as nonlinear SVMs or artificial neural networks, which can let interactions between features and nonlinear functions thereof drive the prediction). Our strategy was to use the SVM classifier with 3-fold cross-validation to classify correct and incorrect memory trials of all memory conditions, using the SEEG contact as features. For each participant, the model is trained only on data 2/3 of the trials to predict whether each trial in the remaining 1/3 set of trials is correct or incorrect. The procedure is repeated 3 further times to estimate the classification performance across the full set folds. As all participants had more correct than incorrect trials for all memory conditions, we made a random selection of the correct trials (to match the number of incorrect trials for each condition) to train and test the classifier. Then, we repeated this analysis 100 times with 100 different random selection of correct trials for each participant. SVM’s performance was evaluated using the output of the 100 models (accuracy minus chance) for each subject. For each subject with above chance decoding accuracy, we extracted the features weights (zscore) to evaluate the relative contribution of each feature (SEEG contact) in the classification.

### Phase consistency analysis

We extracted the PAC consistency across trials and participants in the brain regions where the PAC strength was correlated with behavioural performance (see Figs 4A and 5A and S5 Table). For each trial, we extracted the magnitude of gamma oscillations (30 to 90 Hz) as a function of the phase of the theta oscillation (4 Hz; phase divided into 8 bins). We then extracted the intertrial phase locking (PLV) on these signals using PLV functions available in Brainstorm. To identify whether significant preferred coupling phase could be identified, we extracted for each SEEG contact the gamma power for 8 different phase bins of the theta cycle. To define if a preferred coupling phase can be identified across trials and participant for each region, we used LMMs. The variability between participants was modeled by defining by-participant random intercepts. LMMs were performed in R 3.4.1 using the lme4 [78] and car [79] packages. Both fixed and random factors were considered in statistical modeling. Wald chi-squared tests were used for fixed effects in LMM [79]. The fixed effect represents the mean effect across all participants after accounting for variability. We considered the results of the main analyses significant at *p* < .05. When we found a significant main effect, post hoc honest significant difference (HSD) tests were systematically performed using the R emmeans package (emmeans version 1.6.3). *P* values were considered as significant at *p* < .05 and were adjusted for the number of comparisons performed. More precisely, to avoid increased Type I error when multiple comparisons were performed, the *p*-value of the Tukey HSD test was adjusted using the Tukey method for comparing the given number of estimates.

## Supporting information

S1 Fig Brain oscillations displayed with a logarithmic scale for the frequency axis. (**A**) T-values in the time-frequency domain (*t* test relative to baseline −1,000 to 0 before stimulus onset, FDR corrected in time and frequency domains) of SEEG contacts located in the right and left Heschl’s gyrus (displayed on the single subject T1 in the MNI space provided by SPM12) for a trial time window (−1,000 to 6,000 ms) for the condition 6-tone memory load, 2 s retention period (*n* = 5). (**B**) T-values in the time-frequency domain (*t* test relative to baseline −1,000 to 0 before stimulus onset, FDR corrected in time and frequency domains) of SEEG contacts located in the right and left hippocampus (displayed on the single subject T1 in the MNI space provided by SPM12) for a trial time window (−1,000 to 6,000 ms) for the condition 6-tone memory load, 2 s retention period (*n* = 14).
(PDF)

S2 Fig Theta (orange) and gamma (red) magnitude averaged over SEEG contacts located in regions showing increased power relative to baseline during retention presented as a function of task (memory, perception). NS, nonsignificant.
(PDF)

S3 Fig Memory minus perception (the colormap represents the difference in PAC strength between memory and perception trial—note that the contrast is not significant) for the co-modulogram in SEEG contacts that had previously shown an increase in theta and gamma power identified in Fig 1F, retention period). (PDF)

S4 Fig Theta-gamma PAC in the hippocampus and ventral auditory stream correlates with behavior. Left panel: SEEG contacts showing a positive (hot colormap) and negative (blue colormap) relationship between theta-gamma PAC and performance using data from conditions performed by all 16 participants (6 tones encoding 2 s retention and 6 tones encoding 8 s retention). Results are displayed on the single subject T1 in the MNI space provided by SPM12.
(PDF)

S1 Table Regions and coordinates Fig 1D: Heschl’s gyrus. (PDF)

S2 Table Regions and coordinates Fig 1E: Hippocampal regions. (PDF)

S3 Table Regions and coordinates Fig 2B: PAC memory vs. perception L, Left; R, Right; Sup, Superior; Mid, Middle; Inf, Inferior. (PDF)

S4 Table Coordinates of the maximum value (zscore) of the features weights for each participant with significant above chance decoding accuracy—Fig 3C, L, Left; R, Right; Sup, Superior; Mid, Middle; Inf, Inferior; Tri, Triangular. (PDF)

S5 Table Regions and coordinates Fig 4A: Correlation between PAC and IES, L, Left; R, Right; Sup, Superior; Mid, Middle; Inf, Inferior; Oper, Opercular. (PDF)

S6 Table Regions and coordinates Fig 4B: Correlation between PAC and IES. (PDF)

S7 Table Post hoc tests of Fig 5C: Left STS. (PDF)

S8 Table Post hoc tests of Fig 5C: Left IFG. (PDF)

S9 Table Post hoc tests of Fig 5C: Left hippocampus. (PDF)

S10 Table Post hoc tests of Fig 5C: Right ITG. (PDF)

## Abbreviations

HSD: honest significant difference
IES: inverse efficiency score
IFG: inferior frontal gyrus
ITG: inferior temporal gyrus
LMM: linear mixed model
LTM: long-term memory
PAC: phase amplitude coupling
PLV: phase locking value
RT: response time
STM: short-term memory
STS: superior temporal sulcus
SVM: support vector machine
WM: working memory
