# Supplementary material for: Cross-frequency coupling in cortico-hippocampal networks supports the maintenance of sequential auditory information in short-term memory
Source: PLoS Biol. 2024 Mar 5;22(3):e3002512. doi: 10.1371/journal.pbio.3002512 (PMC10914261; doi:10.1371/journal.pbio.3002512)
Supplement: S1 Fig — (A) T-values in the time-frequency domain (t test relative to baseline −1,000 to 0 before stimulus onset, FDR corrected in time and frequency domains) of SEEG contacts located in the right and left Heschl’s gyrus (displayed on the single subject T1 in the MNI space provided by SPM12) for a trial time window (−1,000 to 6,000 ms) for the condition 6-tone memory load, 2 s retention period (n = 5). (B) T-values in the time-frequency domain (t test relative to baseline −1,000 to 0 before stimulus onset, FDR corrected in time and frequency domains) of SEEG contacts located in the right and left hippocampus (displayed on the single subject T1 in the MNI space provided by SPM12) for a trial time window (−1,000 to 6,000 ms) for the condition 6-tone memory load, 2 s retention period (n = 14). (PDF) [file pbio.3002512.s001.pdf]

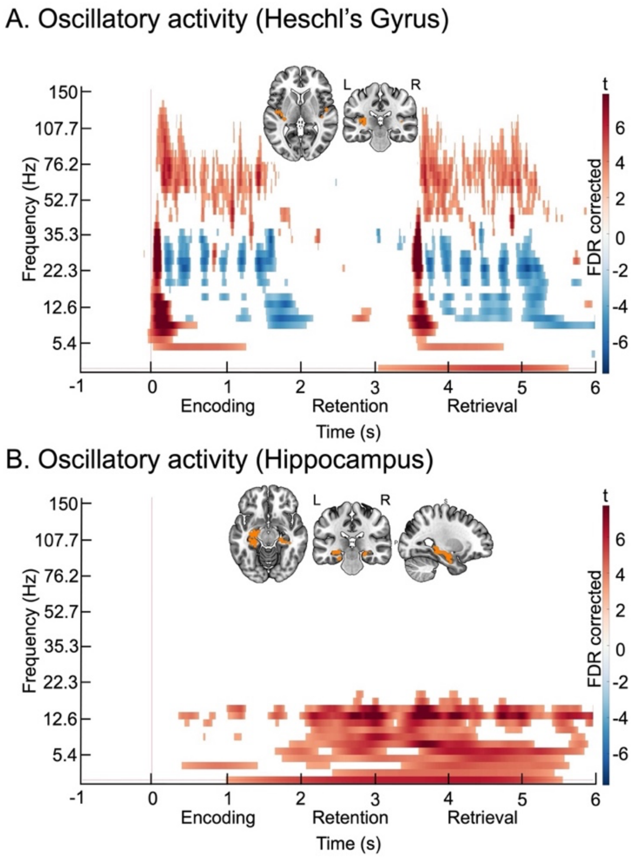

Fig S1: Brain oscillations displayed with a logarithmic scale for the frequency axis. A. T-Values in the Time-Frequency domain (t-test relative to baseline -1000 to 0 before stimulus onset, FDR corrected in time and frequency domains) of SEEG contacts located in the right and left Heschl's gyrus (displayed on the single subject T1 in the MNI space provided by SPM12) for a trial time window (-1000 to 6000 ms) for the condition 6 tone memory load, 2 sec retention period (n=5). B. T-Values in the Time-Frequency domain (t-test relative to baseline -1000 to 0 before stimulus onset, FDR corrected in time and frequency domains) of SEEG contacts located in the right and left Hippocampus (displayed on the single subject T1 in the MNI space provided by SPM12) for a trial time window (-1000 to 6000 ms) for the condition 6 tone memory load, 2 sec retention period (n=14).
