# Supplementary material for: Cross-frequency coupling in cortico-hippocampal networks supports the maintenance of sequential auditory information in short-term memory
Source: PLoS Biol. 2024 Mar 5;22(3):e3002512. doi: 10.1371/journal.pbio.3002512 (PMC10914261; doi:10.1371/journal.pbio.3002512)
Supplement: S2 Fig — NS, nonsignificant. (PDF) [file pbio.3002512.s002.pdf]

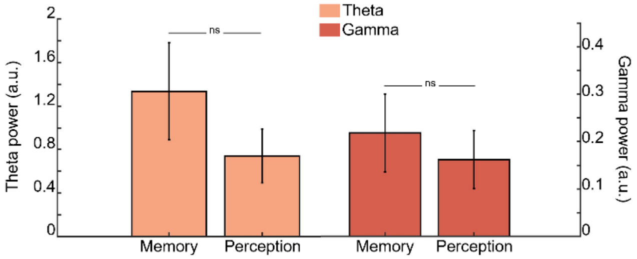

Fig S2: Theta (orange) and gamma (red) magnitude averaged over SEEG contacts located in regions showing increased power relative to baseline during retention presented as a function of task (memory, perception). NS: non-significant.
