# Supplementary material for: Cross-frequency coupling in cortico-hippocampal networks supports the maintenance of sequential auditory information in short-term memory
Source: PLoS Biol. 2024 Mar 5;22(3):e3002512. doi: 10.1371/journal.pbio.3002512 (PMC10914261; doi:10.1371/journal.pbio.3002512)
Supplement: S3 Fig — (PDF) [file pbio.3002512.s003.pdf]

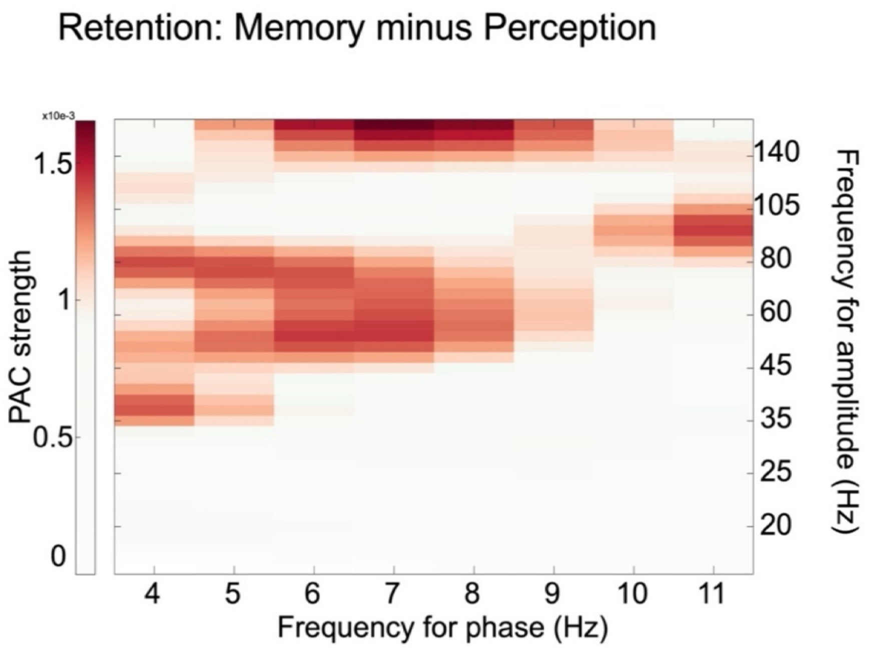

Fig S3: Memory minus Perception (the colormap represents the difference in PAC strength between memory and perception trial -note that the contrast is not significant) for the co-modulogram in SEEG contacts that had previously shown an increase in theta and gamma power identified in Figure 1F, retention period).
