# Supplementary material for: Cross-frequency coupling in cortico-hippocampal networks supports the maintenance of sequential auditory information in short-term memory
Source: PLoS Biol. 2024 Mar 5;22(3):e3002512. doi: 10.1371/journal.pbio.3002512 (PMC10914261; doi:10.1371/journal.pbio.3002512)
Supplement: S4 Fig — Left panel: SEEG contacts showing a positive (hot colormap) and negative (blue colormap) relationship between theta-gamma PAC and performance using data from conditions performed by all 16 participants (6 tones encoding 2 s retention and 6 tones encoding 8 s retention). Results are displayed on the single subject T1 in the MNI space provided by SPM12. (PDF) [file pbio.3002512.s004.pdf]

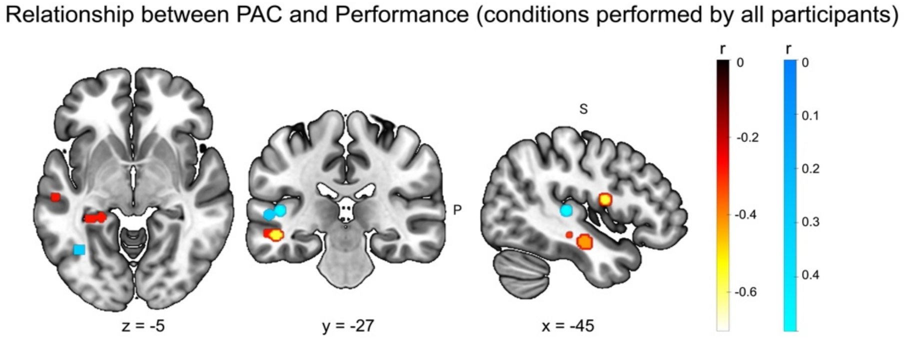

Fig S4: Theta-gamma PAC in the hippocampus and ventral auditory stream correlates with behavior. Left panel: SEEG contacts showing a positive (hot colormap) and negative (blue colormap) relationship between theta-gamma PAC and performance using data from conditions performed by all 16 participants (6 tones encoding; 2sec retention and 6 tones encoding 8s retention). Results are displayed on the single subject T1 in the MNI space provided by SPM12.
