# Supplementary material for: Cross-frequency coupling in cortico-hippocampal networks supports the maintenance of sequential auditory information in short-term memory
Source: PLoS Biol. 2024 Mar 5;22(3):e3002512. doi: 10.1371/journal.pbio.3002512 (PMC10914261; doi:10.1371/journal.pbio.3002512)
Supplement: S1 Table — (PDF) [file pbio.3002512.s005.pdf]

Table S1: regions and coordinates Fig 1D: Heschl's Gyrus

| Coordinates | | | | |
|---|---|---|---|---|
| **X** | **Y** | **Z** | **AAL3** | **Subject** |
| -30 | -27 | 9 | 'Heschl_L' | 2 |
| -38 | -27 | 9 | 'Heschl_L' | 2 |
| -46 | -14 | 6 | 'Heschl_L' | 2 |
| -31 | -27 | 3 | 'Heschl_L' | 3 |
| -36 | -19 | 4 | 'Heschl_L' | 4 |
| -41 | -13 | 2 | 'Heschl_L' | 5 |
| -31 | -25 | 12 | 'Heschl_L' | 8 |
| 50 | -9 | 4 | 'Heschl_R' | 13 |
| 41 | -23 | 7 | 'Heschl_R' | 13 |

| **Subject** | 1 | 2 | 3 | 4 | 5 | 6 | 7 | 8 | 9 | 10 | 11 | 12 | 13 | 14 | 15 | 16 |
|---|---|---|---|---|---|---|---|---|---|---|---|---|---|---|---|---|
| **Contacts** | 0 | 3 | 1 | 1 | 1 | 0 | 0 | 1 | 0 | 0 | 0 | 0 | 2 | 0 | 0 | 0 |
