# Supplementary material for: Cross-frequency coupling in cortico-hippocampal networks supports the maintenance of sequential auditory information in short-term memory
Source: PLoS Biol. 2024 Mar 5;22(3):e3002512. doi: 10.1371/journal.pbio.3002512 (PMC10914261; doi:10.1371/journal.pbio.3002512)
Supplement: S2 Table — (PDF) [file pbio.3002512.s006.pdf]

Table S2: regions and coordinates Fig 1E: Hippocampal regions

Coordinates				
X	Y	Z	AAL3	Subject
17	-7	-20	'ParaHippocampal_R'	1
24	-7	-20	'Hippocampus_R'	1
31	-6	-19	'ParaHippocampal_L'	1
-22	-8	-28	'ParaHippocampal_L'	1
-29	-8	-27	'Hippocampus_R'	1
16	-30	-10	'Hippocampus_R'	1
32	-30	-10	'ParaHippocampal_L'	1
-23	-8	-26	'Hippocampus_L'	1
-22	-31	-10	'Hippocampus_L'	1
-30	-32	-11	'Hippocampus_L'	1
-22	-15	-13	'Hippocampus_L'	1
-29	-16	-14	'Hippocampus_L'	1
-23	-37	-1	'Hippocampus_L'	1
-31	-38	-1	'Hippocampus_L'	1
-25	-17	-11	'Hippocampus_L'	2
-23	-10	-26	'ParaHippocampal_L'	2
-18	-11	-15	'Hippocampus_L'	3
-26	-10	-15	'Hippocampus_L'	3
-22	-16	-15	'Hippocampus_L'	3
-30	-16	-15	'Hippocampus_L'	3
-19	-13	-21	'Hippocampus_L'	3
-27	-13	-21	'Hippocampus_L'	3
-22	-31	-7	'Hippocampus_L'	3
-30	-31	-8	'Hippocampus_L'	3
-21	-11	-18	'Hippocampus_L'	4
-28	-11	-18	'Hippocampus_L'	4
-35	-12	-17	'Hippocampus_L'	4
-25	-16	-14	'Hippocampus_L'	5
-32	-16	-14	'Hippocampus_L'	5
-25	-33	-9	'Hippocampus_L'	5
-32	-33	-9	'Hippocampus_L'	5
21	-11	-21	'Hippocampus_R'	6
28	-11	-22	'Hippocampus_R'	6
35	-11	-23	'Hippocampus_R'	6
25	-32	-8	'Hippocampus_R'	6
32	-32	-9	'Hippocampus_R'	6
39	-32	-10	'Hippocampus_R'	6
23	-18	-25	'ParaHippocampal_R'	6
30	-18	-26	'ParaHippocampal_R'	6
25	-42	-5	'ParaHippocampal_R'	6
32	-42	-6	'ParaHippocampal_R'	6
-18	-13	-24	'ParaHippocampal_L'	8

-22	-20	-11	‘Hippocampus_L’	8
-30	-20	-10	‘Hippocampus_L’	8
-20	-33	-3	‘Hippocampus_L’	8
-28	-33	-3	‘Hippocampus_L’	8
-15	-37	-8	‘ParaHippocampal_L’	8
-23	-37	-7	‘ParaHippocampal_L’	8
-20	-5	-30	‘ParaHippocampal_L’	8
-25	-31	-7	‘Hippocampus_L’	9
24	-42	4	‘Hippocampus_R’	10
-26	-26	-10	‘Hippocampus_L’	11
-34	-26	-11	‘Hippocampus_L’	11
-23	-28	-22	‘ParaHippocampal_L’	11
-24	-38	-10	‘ParaHippocampal_L’	11
-24	-38	-10	‘ParaHippocampal_L’	11
-27	-8	-18	‘Hippocampus_L’	12
-25	-34	-9	‘Hippocampus_L’	12
-33	-34	-9	‘Hippocampus_L’	12
22	-8	-23	‘Hippocampus_R’	13
30	-7	-23	‘Hippocampus_R’	13
24	-13	-27	‘ParaHippocampal_R’	13
23	-26	-13	‘Hippocampus_R’	13
-18	-37	-4	‘ParaHippocampal_L’	13
19	-43	-4	‘ParaHippocampal_R’	13
27	-43	-5	‘ParaHippocampal_R’	13
36	-42	-5	‘ParaHippocampal_R’	13
-23	-29	-10	‘Hippocampus_L’	14
-30	-29	-10	‘Hippocampus_L’	14
-21	-16	-26	‘ParaHippocampal_L’	14
-18	-11	-19	‘Hippocampus_L’	16
-22	-28	-21	‘ParaHippocampal_L’	16

**Subject**	1	2	3	4	5	6	7	8	9	10	11	12	13	14	15	16
**Contacts**	14	2	8	3	4	10	0	8	1	1	5	3	8	3	0	2
