# Supplementary material for: Cross-frequency coupling in cortico-hippocampal networks supports the maintenance of sequential auditory information in short-term memory
Source: PLoS Biol. 2024 Mar 5;22(3):e3002512. doi: 10.1371/journal.pbio.3002512 (PMC10914261; doi:10.1371/journal.pbio.3002512)
Supplement: S3 Table — (PDF) [file pbio.3002512.s007.pdf]

Table S3: regions and coordinates Fig 2B: PAC memory vs. Perception L, Left; R, Right; Sup, Superior; Mid, Middle; Inf, Inferior.

**Coordinates**				
**X**	**Y**	**Z**	**AAL3**	**Subject**
-31	-3	-17	‘Hippocampus_L’	1
58	-10	-25	'Temporal_Mid_R'	6
52	-17	-28	'Temporal_Inf_R'	6
58	-10	-25	'Temporal_Mid_R'	6
45	-17	-27	'Temporal_Inf_R'	6
52	-17	-28	'Temporal_Inf_R'	6
49	-63	-14	'Temporal_Inf_R'	6
56	-63	-15	'Temporal_Inf_R'	6
38	-7	-5	'Insula_R'	6
45	-7	-6	'Temporal_Sup_R'	6
53	-7	-6	'Temporal_Sup_R'	6
52	-67	7	'Temporal_Mid_R'	6
56	-67	6	'Temporal_Mid_R'	6
-30	-4	-25	‘Hippocampus_L’	11

**Subject**	1	2	3	4	5	6	7	8	9	10	11	12	13	14	15	16
**Contacts**	1	0	0	0	0	15	0	0	0	0	1	0	0	0	0	0
