# Supplementary material for: Cross-frequency coupling in cortico-hippocampal networks supports the maintenance of sequential auditory information in short-term memory
Source: PLoS Biol. 2024 Mar 5;22(3):e3002512. doi: 10.1371/journal.pbio.3002512 (PMC10914261; doi:10.1371/journal.pbio.3002512)
Supplement: S4 Table — (PDF) [file pbio.3002512.s008.pdf]

Table S4: Coordinates of the maximum value (zscore) of the features weights for each participant with significant above chance decoding accuracy– Fig 3C, L, Left; R, Right; Sup, Superior; Mid, Middle; Inf, Inferior; Tri, Triangular

Coordinates				
**X**	**Y**	**Z**	**AAL3**	**Subject**
-30	-32	-11	'Hippocampus_L'	1
-67	-13	7	'Temporal_Sup_L'	2
-34	-28	-3	'Hippocampus_L'	4
-47	-71	-4	'Occipital_Inf_L'	5
-30	21	51	'Frontal_Mid_2_L'	7
-49	-39	-5	'Temporal_Mid_L'	8
36	11	-39	'Temporal_Pole_Mid_R'	9
51	-33	15	'Temporal_Sup_R'	10
-56	-49	-18	'Temporal_Inf_L'	11
23	-26	-13	'Hippocampus_R'	13
-41	23	14	'Frontal_Inf_Tri_L'	15
-44	-17	5	'Temporal_Sup_L'	16

**Subject**	1	2	3	4	5	6	7	8	9	10	11	12	13	14	15	16
**Contacts**	1	1	0	1	1	0	1	1	1	1	1	0	1	0	1	1
