# Supplementary material for: Cross-frequency coupling in cortico-hippocampal networks supports the maintenance of sequential auditory information in short-term memory
Source: PLoS Biol. 2024 Mar 5;22(3):e3002512. doi: 10.1371/journal.pbio.3002512 (PMC10914261; doi:10.1371/journal.pbio.3002512)
Supplement: S5 Table — (PDF) [file pbio.3002512.s009.pdf]

Table S5: regions and coordinates Fig 4A: correlation between PAC and IES, L, Left; R, Right; Sup, Superior; Mid, Middle; Inf, Inferior; Oper, Opercular

**Coordinates**				
**X**	**Y**	**Z**	**AAL3**	**Subject**
-22	-8	-28	'ParaHippocampal_L'	1
-19	-3	-15	'Hippocampus_L'	1
-31	-3	-17	Hippocampus_L	1
65	-10	-26	'Temporal_Mid_R'	6
-21	-3	-20	'Hippocampus_L'	9
57	-5	-23	'Temporal_Mid_R'	13
-39	-4	18	'Insula_L'	14
-47	-1	15	'Frontal_Inf_Oper_L'	15

**Subject**	1	2	3	4	5	6	7	8	9	10	11	12	13	14	15	16
**Contacts**	3	0	0	0	0	1	0	0	1	0	0	0	1	1	1	0
