# Supplementary material for: Cross-frequency coupling in cortico-hippocampal networks supports the maintenance of sequential auditory information in short-term memory
Source: PLoS Biol. 2024 Mar 5;22(3):e3002512. doi: 10.1371/journal.pbio.3002512 (PMC10914261; doi:10.1371/journal.pbio.3002512)
Supplement: S6 Table — (PDF) [file pbio.3002512.s010.pdf]

Table S6: regions and coordinates Fig 4B: correlation between PAC and IES

Coordinates				
**X**	**Y**	**Z**	**AAL3**	**Subject**
-31	-27	3	'Heschl_L'	3
-31	-28	-2	'Heschl_L'	6
-47	-29	7	‘Temporal_Sup_L’	9
65	-10	-26	'Temporal_Sup_L'	11

**Subject**	1	2	3	4	5	6	7	8	9	10	11	12	13	14	15	16
**Contacts**	0	0	1	0	0	1	0	0	1	0	1	0	0	0	0	0
