# Supplementary material for: Cross-frequency coupling in cortico-hippocampal networks supports the maintenance of sequential auditory information in short-term memory
Source: PLoS Biol. 2024 Mar 5;22(3):e3002512. doi: 10.1371/journal.pbio.3002512 (PMC10914261; doi:10.1371/journal.pbio.3002512)
Supplement: S7 Table — (PDF) [file pbio.3002512.s011.pdf]

Table S7: Post-Hoc tests of Fig 5C: left STS

contrast			estimate	SE	df	lower.CL	upper.CL	t.ratio	p.value
-π	-	-3π/4	-0.025598	0.00976	759	-0.05527	0.00407	-2.622	0.1495
-π	<	-π/2	-0.052536	0.00976	759	-0.08221	-0.02286	-5.381	<.0001
-π	-	-π/4	-0.024917	0.00976	759	-0.05459	0.00475	-2.552	0.1754
-π	-	0	-0.004618	0.00976	759	-0.03429	0.02505	-0.473	0.9998
-π	-	π/4	-0.006435	0.00976	759	-0.03611	0.02324	-0.659	0.9979
-π	-	π/2	0.002345	0.00976	759	-0.02733	0.03202	0.240	1.0000
-π	-	3π/4	-0.002636	0.00976	759	-0.03231	0.02704	-0.270	1.0000
-3π/4	-	-π/2	-0.026938	0.00976	759	-0.05661	0.00273	-2.759	0.1070
-3π/4	-	-π/4	0.000681	0.00976	759	-0.02899	0.03035	0.070	1.0000
-3π/4	-	0	0.020980	0.00976	759	-0.00869	0.05065	2.149	0.3843
-3π/4	-	π/4	0.019163	0.00976	759	-0.01051	0.04883	1.963	0.5079
-3π/4	-	π/2	0.027943	0.00976	759	-0.00173	0.05761	2.862	0.0819
-3π/4	-	3π/4	0.022962	0.00976	759	-0.00671	0.05263	2.352	0.2670
-π/2	-	-π/4	0.027619	0.00976	759	-0.00205	0.05729	2.829	0.0894
-π/2	>	0	0.047918	0.00976	759	0.01825	0.07759	4.908	<.0001
-π/2	>	π/4	0.046101	0.00976	759	0.01643	0.07577	4.722	0.0001
-π/2	>	π/2	0.054881	0.00976	759	0.02521	0.08455	5.622	<.0001
-π/2	>	3π/4	0.049900	0.00976	759	0.02023	0.07957	5.111	<.0001
-π/4	-	0	0.020299	0.00976	759	-0.00937	0.04997	2.079	0.4293
-π/4	-	π/4	0.018482	0.00976	759	-0.01119	0.04815	1.893	0.5560
-π/4	-	π/2	0.027262	0.00976	759	-0.00241	0.05693	2.793	0.0983
-π/4	-	3π/4	0.022281	0.00976	759	-0.00739	0.05195	2.282	0.3047
0	-	π/4	-0.001817	0.00976	759	-0.03149	0.02785	-0.186	1.0000
0	-	π/2	0.006963	0.00976	759	-0.02271	0.03663	0.713	0.9966
0	-	3π/4	0.001982	0.00976	759	-0.02769	0.03165	0.203	1.0000
π/4	-	π/2	0.008780	0.00976	759	-0.02089	0.03845	0.899	0.9861
π/4	-	3π/4	0.003799	0.00976	759	-0.02587	0.03347	0.389	0.9999
π/2	-	3π/4	-0.004981	0.00976	759	-0.03465	0.02469	-0.510	0.9996
