# Supplementary material for: Cross-frequency coupling in cortico-hippocampal networks supports the maintenance of sequential auditory information in short-term memory
Source: PLoS Biol. 2024 Mar 5;22(3):e3002512. doi: 10.1371/journal.pbio.3002512 (PMC10914261; doi:10.1371/journal.pbio.3002512)
Supplement: S8 Table — (PDF) [file pbio.3002512.s012.pdf]

Table S8: Post-Hoc tests of Fig 5C: left IFG

contrast			estimate	SE	df	lower.CL	upper.CL	t.ratio	p.value
-π	-	-3π/4	0.009416	0.0081	887	-0.01520	0.03403	1.162	0.9423
-π	-	-π/2	-0.003710	0.0081	887	-0.02833	0.02091	-0.458	0.9998
-π	-	-π/4	-0.012399	0.0081	887	-0.03702	0.01222	-1.530	0.7910
-π	-	0	-0.021794	0.0081	887	-0.04641	0.00282	-2.690	0.1270
-π	-	π/4	-0.011606	0.0081	887	-0.03622	0.01301	-1.432	0.8421
-π	-	π/2	-0.003573	0.0081	887	-0.02819	0.02104	-0.441	0.9999
-π	-	3π/4	-0.006821	0.0081	887	-0.03144	0.01780	-0.842	0.9906
-3π/4	-	-π/2	-0.013126	0.0081	887	-0.03774	0.01149	-1.620	0.7382
-3π/4	-	-π/4	-0.021815	0.0081	887	-0.04643	0.00280	-2.692	0.1262
-3π/4	<	0	-0.031210	0.0081	887	-0.05583	-0.00659	-3.852	0.0031
-3π/4	-	π/4	-0.021023	0.0081	887	-0.04564	0.00359	-2.595	0.1592
-3π/4	-	π/2	-0.012989	0.0081	887	-0.03761	0.01163	-1.603	0.7485
-3π/4	-	3π/4	-0.016237	0.0081	887	-0.04085	0.00838	-2.004	0.4797
-π/2	-	-π/4	-0.008689	0.0081	887	-0.03331	0.01593	-1.072	0.9624
-π/2	-	0	-0.018084	0.0081	887	-0.04270	0.00653	-2.232	0.3336
-π/2	-	π/4	-0.007897	0.0081	887	-0.03251	0.01672	-0.975	0.9779
-π/2	-	π/2	0.000137	0.0081	887	-0.02448	0.02475	0.017	1.0000
-π/2	-	3π/4	-0.003111	0.0081	887	-0.02773	0.02151	-0.384	0.9999
-π/4	-	0	-0.009395	0.0081	887	-0.03401	0.01522	-1.160	0.9430
-π/4	-	π/4	0.000792	0.0081	887	-0.02382	0.02541	0.098	1.0000
-π/4	-	π/2	0.008826	0.0081	887	-0.01579	0.03344	1.089	0.9591
-π/4	-	3π/4	0.005578	0.0081	887	-0.01904	0.03019	0.688	0.9973
0	-	π/4	0.010187	0.0081	887	-0.01443	0.03480	1.257	0.9141
0	-	π/2	0.018221	0.0081	887	-0.00640	0.04284	2.249	0.3237
0	-	3π/4	0.014973	0.0081	887	-0.00964	0.03959	1.848	0.5873
π/4	-	π/2	0.008033	0.0081	887	-0.01658	0.03265	0.991	0.9757
π/4	-	3π/4	0.004785	0.0081	887	-0.01983	0.02940	0.591	0.9990
π/2	-	3π/4	-0.003248	0.0081	887	-0.02786	0.02137	-0.401	0.9999
