# Supplementary material for: Cross-frequency coupling in cortico-hippocampal networks supports the maintenance of sequential auditory information in short-term memory
Source: PLoS Biol. 2024 Mar 5;22(3):e3002512. doi: 10.1371/journal.pbio.3002512 (PMC10914261; doi:10.1371/journal.pbio.3002512)
Supplement: S9 Table — (PDF) [file pbio.3002512.s013.pdf]

Table S9: Post-Hoc tests of Fig 5C: left hippocampus

contrast			estimate	SE	df	lower.CL	upper.CL	t.ratio	p.value
-π	-	-3π/4	-0.01122	0.0058	2327	-0.028832	6.38e-03	-1.934	0.5275
-π	<	-π/2	-0.02788	0.0058	2327	-0.045487	-1.03e-02	-4.804	<.0001
-π	<	-π/4	-0.03235	0.0058	2327	-0.049957	-1.47e-02	-5.574	<.0001
-π	<	0	-0.01770	0.0058	2327	-0.035304	-8.94e-05	-3.049	0.0478
-π	-	π/4	0.00482	0.0058	2327	-0.012788	2.24e-02	0.830	0.9914
-π	-	π/2	0.00872	0.0058	2327	-0.008889	2.63e-02	1.502	0.8066
-π	-	3π/4	0.00651	0.0058	2327	-0.011101	2.41e-02	1.121	0.9524
-3π/4	-	-π/2	-0.01666	0.0058	2327	-0.034263	9.52e-04	-2.870	0.0793
-3π/4	<	-π/4	-0.02113	0.0058	2327	-0.038732	-3.52e-03	-3.640	0.0068
-3π/4	-	0	-0.00647	0.0058	2327	-0.024079	1.11e-02	-1.115	0.9537
-3π/4	-	π/4	0.01604	0.0058	2327	-0.001563	3.37e-02	2.764	0.1046
-3π/4	>	π/2	0.01994	0.0058	2327	0.002335	3.75e-02	3.436	0.0139
-3π/4	>	3π/4	0.01773	0.0058	2327	0.000124	3.53e-02	3.055	0.0470
-π/2	-	-π/4	-0.00447	0.0058	2327	-0.022077	1.31e-02	-0.770	0.9946
-π/2	-	0	0.01018	0.0058	2327	-0.007424	2.78e-02	1.755	0.6511
-π/2	>	π/4	0.03270	0.0058	2327	0.015092	5.03e-02	5.634	<.0001
-π/2	>	π/2	0.03660	0.0058	2327	0.018991	5.42e-02	6.306	<.0001
-π/2	>	3π/4	0.03439	0.0058	2327	0.016779	5.20e-02	5.925	<.0001
-π/4	-	0	0.01465	0.0058	2327	-0.002954	3.23e-02	2.525	0.1854
-π/4	>	π/4	0.03717	0.0058	2327	0.019562	5.48e-02	6.404	<.0001
-π/4	>	π/2	0.04107	0.0058	2327	0.023461	5.87e-02	7.076	<.0001
-π/4	>	3π/4	0.03886	0.0058	2327	0.021249	5.65e-02	6.695	<.0001
0	>	π/4	0.02252	0.0058	2327	0.004909	4.01e-02	3.879	0.0027
0	>	π/2	0.02641	0.0058	2327	0.008808	4.40e-02	4.551	0.0002
0	>	3π/4	0.02420	0.0058	2327	0.006596	4.18e-02	4.170	0.0008
π/4	-	π/2	0.00390	0.0058	2327	-0.013708	2.15e-02	0.672	0.9977
π/4	-	3π/4	0.00169	0.0058	2327	-0.015920	1.93e-02	0.291	1.0000
π/2	-	3π/4	-0.00221	0.0058	2327	-0.019819	1.54e-02	-0.381	0.9999
