# Supplementary material for: Cross-frequency coupling in cortico-hippocampal networks supports the maintenance of sequential auditory information in short-term memory
Source: PLoS Biol. 2024 Mar 5;22(3):e3002512. doi: 10.1371/journal.pbio.3002512 (PMC10914261; doi:10.1371/journal.pbio.3002512)
Supplement: S10 Table — (PDF) [file pbio.3002512.s014.pdf]

Table S10: Post-Hoc tests of Figure 5C: right ITG

contrast			estimate	SE	df	lower.CL	upper.CL	t.ratio	p.value
-π	-	-3π/4	-0.004626	0.00904	1175	-0.03206	0.022807	-0.512	0.9996
-π	-	-π/2	-0.020952	0.00904	1175	-0.04839	0.006482	-2.319	0.2840
-π	<	-π/4	-0.029038	0.00904	1175	-0.05647	-0.001604	-3.214	0.0292
-π	<	0	-0.031454	0.00904	1175	-0.05889	-0.004021	-3.481	0.0121
-π	<	π/4	-0.028866	0.00904	1175	-0.05630	-0.001432	-3.195	0.0310
-π	-	π/2	-0.020170	0.00904	1175	-0.04760	0.007264	-2.232	0.3330
-π	-	3π/4	-0.005370	0.00904	1175	-0.03280	0.022063	-0.594	0.9989
-3π/4	-	-π/2	-0.016325	0.00904	1175	-0.04376	0.011108	-1.807	0.6155
-3π/4	-	-π/4	-0.024412	0.00904	1175	-0.05185	0.003022	-2.702	0.1229
-3π/4	-	0	-0.026828	0.00904	1175	-0.05426	0.000606	-2.969	0.0606
-3π/4	-	π/4	-0.024240	0.00904	1175	-0.05167	0.003194	-2.683	0.1288
-3π/4	-	π/2	-0.015543	0.00904	1175	-0.04298	0.011891	-1.720	0.6740
-3π/4	-	3π/4	-0.000744	0.00904	1175	-0.02818	0.026690	-0.082	1.0000
-π/2	-	-π/4	-0.008086	0.00904	1175	-0.03552	0.019348	-0.895	0.9865
-π/2	-	0	-0.010503	0.00904	1175	-0.03794	0.016931	-1.162	0.9423
-π/2	-	π/4	-0.007914	0.00904	1175	-0.03535	0.019520	-0.876	0.9881
-π/2	-	π/2	0.000782	0.00904	1175	-0.02665	0.028216	0.087	1.0000
-π/2	-	3π/4	0.015581	0.00904	1175	-0.01185	0.043015	1.725	0.6712
-π/4	-	0	-0.002416	0.00904	1175	-0.02985	0.025017	-0.267	1.0000
-π/4	-	π/4	0.000172	0.00904	1175	-0.02726	0.027606	0.019	1.0000
-π/4	-	π/2	0.008868	0.00904	1175	-0.01857	0.036302	0.982	0.9770
-π/4	-	3π/4	0.023668	0.00904	1175	-0.00377	0.051101	2.620	0.1498
0	-	π/4	0.002588	0.00904	1175	-0.02485	0.030022	0.286	1.0000
0	-	π/2	0.011285	0.00904	1175	-0.01615	0.038719	1.249	0.9169
0	-	3π/4	0.026084	0.00904	1175	-0.00135	0.053518	2.887	0.0761
π/4	-	π/2	0.008697	0.00904	1175	-0.01874	0.036130	0.963	0.9795
π/4	-	3π/4	0.023496	0.00904	1175	-0.00394	0.050929	2.601	0.1566
π/2	-	3π/4	0.014799	0.00904	1175	-0.01263	0.042233	1.638	0.7271
